# An Interventional Response Phenotyping Study in Chronic Low Back Pain: Protocol for a Mechanistic Randomized Controlled Trial

**DOI:** 10.1093/pm/pnad005

**Published:** 2023-01-27

**Authors:** Afton L Hassett, David A Williams, Richard E Harris, Steven E Harte, Chelsea M Kaplan, Andrew Schrepf, Anna L Kratz, Chad M Brummett, Kelley M Kidwell, Alexander Tsodikov, Sana Shaikh, Susan L Murphy, Remy Lobo, Anthony King, Todd Favorite, Laura Fisher, Goodarz M Golmirzaie, David J Kohns, Jill R Schneiderhan, Ishtiaq Mawla, Eric Ichesco, Jenna McAfee, Ronald A Wasserman, Elizabeth Banner, Kathy A Scott, Courtney Cole, Daniel J Clauw

**Affiliations:** Department of Anesthesiology, University of Michigan, Ann Arbor, MI 48106, United States; Department of Anesthesiology, University of Michigan, Ann Arbor, MI 48106, United States; Department of Anesthesiology, University of Michigan, Ann Arbor, MI 48106, United States; Department of Anesthesiology, University of Michigan, Ann Arbor, MI 48106, United States; Department of Anesthesiology, University of Michigan, Ann Arbor, MI 48106, United States; Department of Anesthesiology, University of Michigan, Ann Arbor, MI 48106, United States; Department of Physical Medicine and Rehabilitation, University of Michigan, Ann Arbor, MI 48109, United States; Department of Anesthesiology, University of Michigan, Ann Arbor, MI 48106, United States; Department of Anesthesiology, University of Michigan, Ann Arbor, MI 48106, United States; Department of Biostatistics, School of Public Health, University of Michigan, Ann Arbor, MI 48109, United States; Department of Anesthesiology, University of Michigan, Ann Arbor, MI 48106, United States; Department of Physical Medicine and Rehabilitation, University of Michigan, Ann Arbor, MI 48109, United States; Department of Radiology, University of Michigan, Ann Arbor, MI 48109, United States; Department of Psychiatry, Ohio State University, Columbus, OH 43210, United States; Department of Psychiatry, University of Michigan, Ann Arbor, MI 48109, United States; Department of Physical Medicine and Rehabilitation, University of Michigan, Ann Arbor, MI 48109, United States; Department of Anesthesiology, University of Michigan, Ann Arbor, MI 48106, United States; Department of Physical Medicine and Rehabilitation, University of Michigan, Ann Arbor, MI 48109, United States; Department of Family Medicine, University of Michigan, Ann Arbor, MI 48109, United States; Department of Anesthesiology, University of Michigan, Ann Arbor, MI 48106, United States; Department of Anesthesiology, University of Michigan, Ann Arbor, MI 48106, United States; Department of Anesthesiology, University of Michigan, Ann Arbor, MI 48106, United States; Department of Anesthesiology, University of Michigan, Ann Arbor, MI 48106, United States; Department of Anesthesiology, University of Michigan, Ann Arbor, MI 48106, United States; Department of Anesthesiology, University of Michigan, Ann Arbor, MI 48106, United States; Department of Anesthesiology, University of Michigan, Ann Arbor, MI 48106, United States; Department of Anesthesiology, University of Michigan, Ann Arbor, MI 48106, United States

## Abstract

Evidence-based treatments for chronic low back pain (cLBP) typically work well in only a fraction of patients, and at present there is little guidance regarding what treatment should be used in which patients. Our central hypothesis is that an interventional response phenotyping study can identify individuals with different underlying mechanisms for their pain who thus respond differentially to evidence-based treatments for cLBP. Thus, we will conduct a randomized controlled Sequential, Multiple Assessment, Randomized Trial (SMART) design study in cLBP with the following three aims. Aim 1: Perform an interventional response phenotyping study in a cohort of cLBP patients (n = 400), who will receive a sequence of interventions known to be effective in cLBP. For 4 weeks, all cLBP participants will receive a web-based pain self-management program as part of a run-in period, then individuals who report no or minimal improvement will be randomized to: a) mindfulness-based stress reduction, b) physical therapy and exercise, c) acupressure self-management, and d) duloxetine. After 8 weeks, individuals who remain symptomatic will be re-randomized to a different treatment for an additional 8 weeks. Using those data, we will identify the subsets of participants that respond to each treatment. In Aim 2, we will show that currently available, clinically derived measures, can predict differential responsiveness to the treatments. In Aim 3, a subset of participants will receive deeper phenotyping (n = 160), to identify new experimental measures that predict differential responsiveness to the treatments, as well as to infer mechanisms of action. Deep phenotyping will include functional neuroimaging, quantitative sensory testing, measures of inflammation, and measures of autonomic tone.

## Introduction

Chronic low back pain (cLBP) affects an estimated 42 million Americans and is associated with greater healthcare utilization, higher rates of unemployment, worse sleep and more depression compared to those without cLBP.^1^ At present there are data suggesting a variety of structural/mechanical, neural, psychological, cognitive, behavioral, social, and economic contributors to cLBP. Not surprisingly, without a clear understanding of the causes of cLBP, treatment effectiveness has suffered, and many individuals fail to get adequate pain relief. Further, concerns about the opioid epidemic, as well as an aging population that boosts the prevalence of cLBP,^2^ emphasize the critical need to advance how we conceptualize and treat cLBP.

The biopsychosocial model of chronic pain acknowledges the complex set of pathogenic contributors to the etiology and maintenance of cLBP. The Back Pain Consortium (BACPAC) Research Program has chosen to address the many facets of the biopsychosocial model in a comprehensive and unbiased manner and provides an integrated translational approach to identifying both the underlying mechanisms operative in cLBP, as well as the treatments that work on those underlying mechanisms. The University of Michigan BACPAC Interdisciplinary Mechanistic Research Center (MRC) is one of three MRCs within the consortium. Each site conducts an independent study, as well as a separate collaborative trial that supports the overall BACPAC Research Program. All sites collect common data elements that in addition to site specific data will be combined to create a robust data set with the goal of enhancing our understanding about who responds best to what treatment.

The most widely used treatment options for cLBP typically include a combination of medication and surgical or interventional procedures, with the goal of relieving pain and restoring function. While medications can be modestly beneficial for some patients with chronic pain,^3–6^ behavioral interventions such as cognitive-behavioral therapy (CBT) have demonstrated similar, albeit modest, effects for reducing symptoms.^4^^,^^7–10^ It is now widely accepted that optimal management for cLBP includes treatments that address not just the biological causes of pain, but also the role of psychosocial factors in the development and maintenance of chronic pain.

Given the largely inadequate effects of current treatments, chronic pain remains a serious public health issue and there must be a cultural transformation in how pain is understood, assessed, and treated. One possible explanation for the small effect sizes seen with most treatments for cLBP is that patients are not being adequately matched to appropriate interventions. We hypothesize that an interventional response phenotyping study can identify individuals with different underlying mechanisms for their pain who thus respond differentially to evidence-based interventions for cLBP. To address our hypothesis, we will conduct a single-site Sequential, Multiple Assignment, Randomized Trial (SMART) for the treatment of cLBP.

## Overview and study aims

The first aim of this BACPAC project is to perform an Interventional Response Phenotyping study in a cohort of cLBP patients. Participants will receive a sequence of interventions known to be effective in cLBP. After a 4-week run-in period where all participants have access to an online pain self-management program known as PainGuide, individuals who report no or minimal improvement in their pain (Patient Global Impression of Change [PGIC] score ≥ 2) will be randomized to a series of treatments, including: a) mindfulness-based stress reduction (MBSR, n = 100), b) physical therapy and exercise (PT, n = 100), c) acupressure *m*Health self-management (acupressure, n = 100), or d) duloxetine (n = 100). After 8 weeks, individuals who report no or minimal improvement in their pain will be re-randomized to a different treatment for an additional 8 weeks. Those who are no longer symptomatic will be encouraged to continue the treatment and complete follow-up assessments.

The second aim of our project is to demonstrate that currently available, clinically derived measures, can predict differential responsiveness to the above therapies. We will leverage the SMART noted above to perform the most comprehensive study-to-date of predictors for commonly used cLBP therapies. All patients evaluated in Aim 1 will complete baseline clinical phenotyping that will include the following potential predictors of treatment response: a) demographics, b) questionnaires assessing underlying pain mechanisms, c) ambulatory symptom monitoring, d) extensive psychological assessment using validated patient-reported outcomes, e) structured physical examination, and f) state-of-the-art structural imaging of the back and pelvis.

The third aim of our study is to identify new experimental measures that predict differential responsiveness to each of the above therapies, as well as to infer mechanisms of action for the treatments. A subset of individuals (n = 160) from the larger cohort in Aims 1 and 2 will be asked to participate in an expanded phenotyping study that will include structural and functional brain neuroimaging, quantitative sensory testing (QST), measures of inflammation in blood, and digital measurement of autonomic tone.

## Methods

### Study design

**Figure 1.**
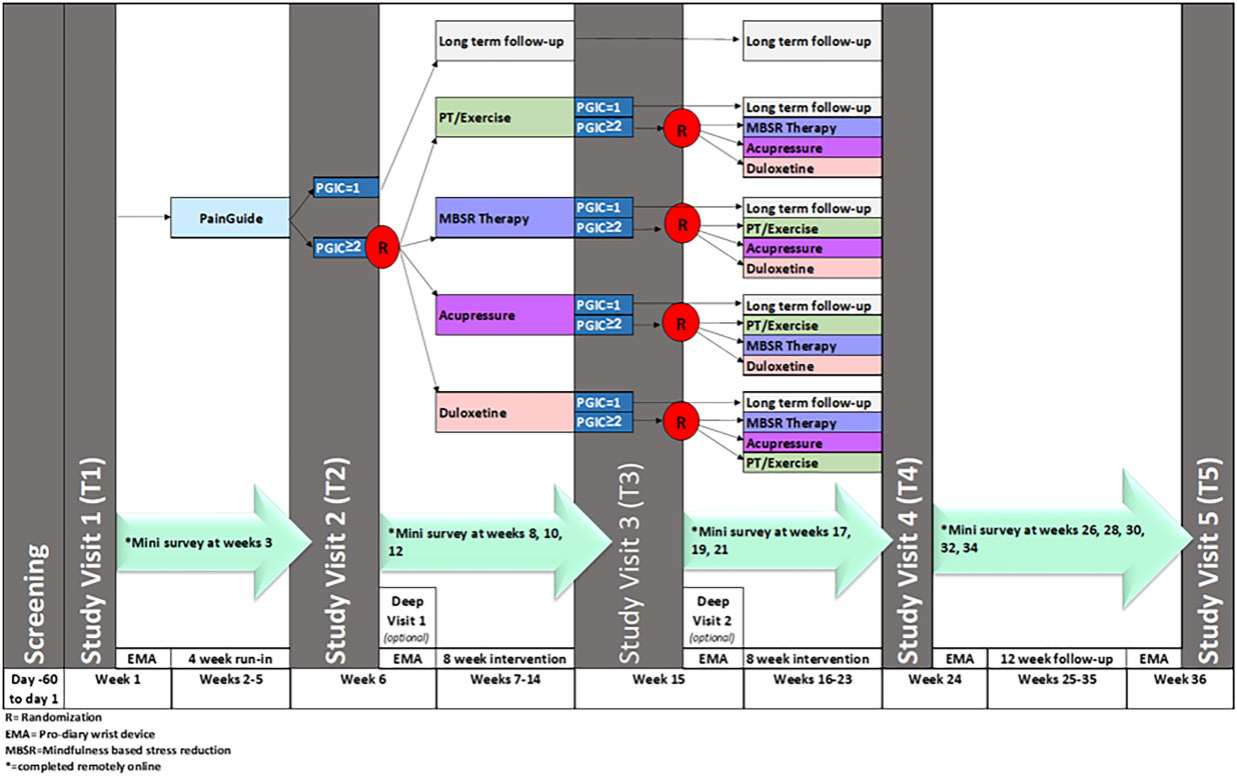
Overview of the study design and assessment plan.

We will conduct a SMART in a cohort of individuals with cLBP. Figure 1 shows an overview of the study design and assessment plan. The proposed SMART will consist of a 4-week run-in period using an online cognitive-behavioral self-management intervention (PainGuide), followed by two 8-week treatment periods. All participants will be followed for approximately nine months. At baseline (Time 1 assessment, T1), all patients will complete informed consent and then undergo a comprehensive baseline phenotyping assessment. After receiving PainGuide for 4 weeks, all participants will complete a light phenotyping at Time 2 assessment (T2) and a subset of these patients (n = 160) will complete an additional deep phenotyping assessment. Those who report no or minimal improvement in their pain (PGIC score ≥ 2) will be randomized to one of the four 8-week long interventions (ie, MBSR, PT, acupressure, or duloxetine). Following the first 8-week treatment period, patients will be reassessed at the Time 3 assessment (T3) using light only or light plus deep assessments (for the subset of 160) and those who report no or minimal improvement in their pain (PGIC ≥ 2) will be re-randomized to receive one of the three treatments they did not receive in the first treatment period. After the second 8-week intervention period, all undergo the light phenotyping follow-up assessment protocol at the Time 4 assessment (T4). Lastly, a final assessment, Time 5 (T5), will take place at 3 months after the scheduled end of the second 8-week treatment period. There will also be a series of 12 “mini” assessments that take place at 2-week intervals in between the regular assessments (T1–T5). Study completion date will be the date of the T5 study visit.

A subset of patients (n = 160) will undergo “deep” phenotyping, with additional testing such as QST, functional magnetic resonance imaging (fMRI), autonomic nervous system (ANS) function assessment, and additional blood collection for basal and stimulated immune markers. There will be two deep phenotyping assessments that take place before and after Treatment 1. This study has been approved by IRBMED at the University of Michigan (HUM# 00180994) and has been registered at ClinicalTrials, gov, NCT 0487057.

### Study participants

Individuals who are ages 25–70 years and are being seen at the University of Michigan Health (UMH) will be invited to participate. A total of 400 participants will be randomized into one of the four treatment arms. When an arm reaches 100 participants, new participants will be randomized into one of the remaining open arms. It is expected that approximately 500 participants will be consented to meet this randomization goal. Recruitment will end when 100 participants have been assigned to each treatment arm.

There will be two sets of inclusion and exclusion criteria. The first set of criteria is for enrollment into the research project for all participants (see Table 1). The second set represents additional inclusion and exclusion criteria that are necessary for the safe and valid conduct of the deep phenotyping protocol (subset n = 160). Deep phenotyping criteria are shown in Table 2. Participants must qualify for and be willing to be randomized into at least three of the four treatments. Contraindications for treatments appear in Table 3.

**Table 1.** Light phenotyping inclusion and exclusion criteria

Inclusion Criteria	Exclusion Criteria
Meet the definition of cLBP described in the NIH Task Force Report on Research Standards for Chronic Low Back Pain. ie, low back pain presents at least six months, and presents more than half of those days. Individuals must have a pain interference score of ≥60 on PROMIS Pain Interference. The normal population mean for pain interference is 50. Participants must be 1 SD above the population mean (≥60) for inclusion. Individuals must be willing and eligible to be randomized to receive at least three of the four proposed treatments.	History of: discitis osteomyelitis (spine infection) or spine tumor ankylosing spondylitis, rheumatoid arthritis, polymyalgia rheumatica, or psoriatic arthritis, lupus cauda equina syndrome or spinal radiculopathy with functional motor deficit Diagnosis of any vertebral fracture in the last 6 months Osteoporosis requiring treatment other than vitamin D and calcium supplements Cancer (History of any bone-related cancer or cancer that metastasized to the bone; currently in treatment for cancer or plan to start treatment in the next 12 months; history of any cancer treatment in the last 24 months) Life expectancy less than 2 years Unable to speak and write English Visual or hearing difficulties that would preclude participation Presence of any history that would preclude scanning in MRI Uncontrolled drug/alcohol addiction Individuals started receiving disability or compensation within the past year, or currently involved in litigation Pregnancy or breastfeeding Individuals on high doses of opioids (over 100 OME per day) Scheduled back surgery, back surgery within the last year, or more than one back surgery in the past. Expecting to receive an injection or surgical procedure within the next year for their cLBP Current/planned (next 2 years) enrollment in another study of a device or investigational drug that would interfere with this study, this may include participation in a blinded trial. Any other diseases or conditions that would make a patient unsuitable for study participation as determined by the site principal investigators.

1. Deyo RA, Dworkin SF, Amtmann D, et al. Report of the NIH Task Force on research standards for chronic low back pain. *J Pain.* 2014; 15(6):569–585.

**Table 2.** Deep phenotyping inclusion and exclusion criteria

Inclusion Criteria	Exclusion Criteria
Right hand dominant Normal visual acuity or correctable (with corrective lenses- glasses or contacts) to at least 20/40 for reading instructions in the MRI and visual sensitivity testing No contraindications to MRI (eg, metal implants) Willingness to refrain from taking any “as needed” medications, including pain medications such as NSAIDs (eg, Motrin, Advil, Aleve), acetaminophen (eg, Tylenol), and opioids, for 8 hours before undergoing neuroimaging and QST Willingness to refrain from alcohol and nicotine on the day of QST and neuroimaging (alcohol and nicotine consumption is allowed after testing is completed) Willingness to refrain from any unusual physical activity or exercise that would cause muscle and/or joint soreness for 48 hours prior to testing (routine exercise or activity that does not lead to soreness is acceptable) Able to lie still on their back for 2 hours during MRI	Severe claustrophobia precluding MRI and evoked pain testing during scanning Diagnosed peripheral neuropathy Current, recent (within the last 6 months), or habitual use of artificial nails or nail enhancements. (Artificial nails can influence pressure pain sensitivity at the thumbnail) BMI > 45 or unable to comfortably fit in the bore of the MRI magnet

**Table 3.** Contraindications to study interventions and MRI

Study Interventions		MRI
Duloxetine	Acupressure	
Medications such as: Lithium Tramadol St. John’s Wort Prochlorperazine (Compazine) Thioridizine Propafenone or Flecanide Ciprofloxacin Linezolid Methylene Blue Cimetidine SSRIs: sertraline paroxetine fluoxetine escitalopram citalopram fluvoxamine SNRIs: venlaxaxine milnacipran duloxetine sibutramine atomoxetine desvenlafaxine levomilnacipran Renal dysfunction: Creatinine Clearance (<30mL/min or End-Stage Renal Failure) Hepatic dysfunction: Liver function tests (LFTs) elevated times 1.5 History of allergy to duloxetine	Currently receiving acupressure or acupuncture through a formal therapy	Presence of any history that would preclude scanning in MRI (ie, known metal foreign objects or implants, history of claustrophobia)
	Mindfulness-based Stress Reduction (MBSR)	
	Current participation in a structured MBSR program	
	Physical Therapy and Exercise	
	Currently receiving any type of structured manual therapy or exercise treatment for low-back pain. Any contraindication for manual therapy and/or participation in an exercise program.	

### Recruitment and screening

We will enroll adults meeting the inclusion and exclusion criteria from UMH outpatient clinics such as the Back & Pain Center and satellite clinical sites, as well as the UM Physical Medicine and Rehabilitation, Family Medicine, and Neurosurgery clinical sites. Patients will also be recruited through online platforms such as Facebook, back pain forums, and umhealthresearch.org, through health fairs and passively with study flyers and email campaigns. Additionally, eligible patients may be identified using the Back & Pain Center new patient database known as APOLO^11^ and by using electronic health record queries. Patients will be contacted by phone or in person and screened using study forms. Interested and eligible patients will then be scheduled for an in-person baseline study visit at the Back & Pain Center or seen virtually.

#### Baseline visit

The baseline visit takes place in person, while follow-up study visits may be a hybrid combining in-person and virtual activities to decrease participant burden. Participants complete informed consent and are screened for meeting deep phenotyping inclusion criteria. All who meet criteria for deep phenotyping are invited to take part in the additional assessments and, for those who agree, appointments are set for neuroimaging if timeslots are available. Most participants are not assessed using any of the deep phenotyping approaches if a neuroimaging visit is not possible. Participants who consented but were later found to be ineligible for the study will be considered screen failures.

Participants complete a battery of questionnaires via web-based Qualtrics electronic data capture system, physical function testing, a structured physical exam, a biomechanical assessment, blood draw, and ambulatory data collection via a wrist-worn accelerometer enhanced with a self-report data collection interface (PRO-diary, CamNTech, Cambridge, UK). Participants later undergo an MRI of the back and pelvis within one week of this initial assessment. The elements of assessment, including the list of validated questionnaires, are briefly described below and depicted in Supplement 1 Schedule of Activities and Associated Data Collection at https://medicine.umich.edu/dept/cpfrc/resources-0. As part of the HEAL initiative, a minimum questionnaire dataset and blood sample are collected, all other assessments are specific to this study. More details appear in Supplement 1, as well.

*Questionnaire assessments.* Validated questionnaires will be used to assess many of the variables of interest. Questionnaires will be completed by participants at all visits (T1–T5) and there will be additional mini-assessments that take place every two weeks throughout the study. The primary outcome measure will be the PROMIS Pain Interference short form score at T3 taken at the conclusion of Treatment 1. While this is not a traditional efficacy or effectiveness trial, the measure will be used to assess response to treatment. Note that the PGIC is used to determine whether there is potential for improvement in pain and is not a primary outcome. The PGIC states, “Since the start of the study (treatment), my overall pain is ….,” with the following response options: 0—Very much improved, 1—Much improved, 2—Minimally improved, 3—No Change, 4—Minimally worse, 5—Much worse, and 6—Very much worse. Lastly, additional treatment-related study surveys will be completed by participants in the MBSR and physical therapy interventions.

*Physical function performance tests*. Three performance tests are included in the baseline phenotyping. The first two performance tests come from the NIH Toolbox measures of motor function, a group of validated assessments that have robust psychometric properties and scoring features.^12^ The first test is the 2-Minute Walk Endurance Test. This test is adapted from the American Thoracic Society’s 6-Minute Walk Test Protocol.^13^ The second test is the 4-Meter Walk Gait Speed Test. This test is adapted from the 4-meter walk test in the Short Physical Performance Battery.^14^ The final test is the Five Time Sit to Stand Test,^15^ which is a valid, reliable measure of physical disability in people with cLBP.^16^^,^^17^

*Biomechanical assessment*. Spine kinematic assessments will be used as an exploratory predictor of treatment response. Data are collected using a multiple wearable custom sensors system attached via harnesses to the back and hips in conjunction with a software platform. This system called Conity (Conity.com) interacts with the patient and guides them in performing a ten-minute standard test that observes spine position ranges as well as maximum dynamic activity in three-dimensional space. The Conity system compares the various motion features of the patients to a normative database to interpret the kinematic information.

*Biospecimens*. Blood serum, whole blood, urine, and saliva will be collected as part of the global BACPAC phenotyping effort that includes DNA, transcriptomics, proteomics, and other “omic” analyses. Participants will rest quietly for several minutes prior to venipuncture. A maximum of 20 mL of blood will be drawn from either arm. Approximately 2 mL of saliva is collected in supplied container (eg, Oragene, DNA Genotek) and stored at room temperature until transfer to −20 or −80°C for future whole genome sequencing. Biospecimens will only be collected at baseline.

*Structural MRI of the back and pelvis*. Patients will be scanned on a 3 T Philips magnet. A routine lumbar spine protocol (sagittal T1, sagittal T2 with and without fat saturation, axial T2 in one or two blocks) will be used. Degenerative changes will be scored according to an MRI scoring sheet developed by BACPAC collaborators. Scoring of the MRI will include the following: a) BMIC (Bone Marrow Intensity Changes or Modic Changes), b) Endplate defects, c) Disc Quality, d) Facet Joints, e) Stenosis.

*Actigraphy and Ecological Momentary Assessment (EMA).* Participants will receive instruction on the use of the PRO-Diary monitor, which the participant will wear on their non-dominant wrist during five separate 7-day “home monitoring” periods, to assess physical activity (objectively measured via accelerometry), sleep, and ecological momentary assessment (EMA; real-time) of mental and physical symptoms. The PRO-Diary has an integrated triaxial microelectromechanical systems accelerometer. The monitor samples data at 50 Hz; for each second it records peak acceleration compared to an immobility threshhold (0.1 g). Values below and immobilitiy thresshold are ignored and all other values are summed over the 15-second epochs to yield scores called “activity counts.”

*Deep phenotyping*. A subset of study participants (n = 160) will undergo deep phenotyping. These participants will have a separate assessment appointment with study personnel at the Chronic Pain and Fatigue Research Center. There they will undergo two visits that take place after T2 and before the commencement of Treatment 1 and after T3 (before randomization to Treatment 2). At the deep phenotyping study visit, participants will undergo structural and functional brain neuroimaging, inflammatory markers assessment, and the assessment autonomic functioning. In addition, participants will undergo a battery of static and dynamic QST assessments. In brief, this includes assessments of a) pressure pain sensitivity measured at the trapezius, thumbnail bed, and lower leg, b) conditioned pain modulation (CPM), c) mechanical temporal summation of pain, d) sensitivity to visual stimulation, and e) tactile acuity measured via a two-point discrimination task. Pressure pain threshold at the trapezius and temporal summation are core data elements that will be collected at all BACPAC MRCs following a harmonized protocol, whereas the remainder of assessments are specific to the University of Michigan MRC. Table 4 depicts key elements of the deep phenotyping. Additional details related to all the deep phenotyping methods can be found in Supplement 2 Deep Phenotyping Methods at https://medicine.umich.edu/dept/cpfrc/resources-0.

**Table 4.** Deep phenotyping assessments for a subset of participants (n = 160).

Quantitative Sensory Testing 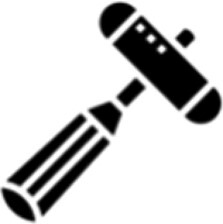	Neuroimaging 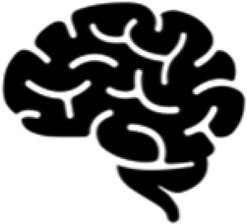	Autonomic Function 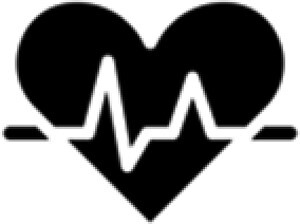	Biospecimens 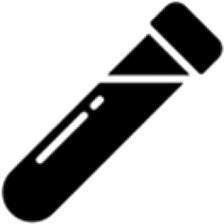
pressure pain sensitivity	structural MRI (T1 and T2)	electrocardiogram	stimulated inflammatory response
conditioned pain modulation	resting state fMRI (pre- and post-evoked pain)	galvanic skin response	cytokines/chemokines
temporal summation	^1^H-MRS of the posterior insula (pre- and post-evoked pain)	respiration	gene expression
visual sensitivity	evoked pain fMRI	skin temperature	sex hormones
two-point discrimination		photoplethysmogram	

### PainGuide run-in period

After the baseline visit, all participants will be assigned to a web-based behavioral pain self-management program known as PainGuide (https://painguide.med.umich.edu/). PainGuide is an online or smartphone accessible website containing education and evidence-based self-management modules for pain. PainGuide offers (a) education about pain, pain mechanisms, types of pain including cLBP, and education about a wide variety of professionally administered pain treatments; (b) a rationale and resources for using a variety of self-management approaches for pain; (c) a system for online monitoring of symptoms and self-management activities; and (d) external resources (eg, current literature, patient advocacy groups) that can support the use of self-management. Participants are instructed to use the website as much as they like. The run-in period was included to help address regression to the mean and orient participants to the study.

### Randomization

Blocked randomization will be used for the randomization schedule. Patients will be assigned to MBSR (n = 100), PT (n = 100), acupressure (n = 100), or duloxetine (n = 100). Participants will be recruited and randomized until all 4 arms of the study have been filled, which will require the recruitment of as many as 500 participants given that we anticipate that 10–20% will respond to the PainGuide self-management intervention, will withdraw, or be lost to follow-up. Randomization will occur at T2 for all participants and T3 for participants with no or minimal improvement in pain after the PainGuide run-in (PGIC ≥ 2). Participants who have a robust improvement in their pain (PGIC < 2) at T3 will not be further randomized to treatment but followed to complete the remaining study visits at T4 and T5. Below is an overview of interventions procedures.

For first stage randomization, five randomization lists will be generated depending on whether participants are eligible for all 4 treatments or eligible for only three of the four treatments. We anticipate 30% of patients are ineligible to receive Duloxetine and very few patients will be ineligible to receive one of the other 3 treatments. We will use random block randomization such that those who were eligible for all four treatments have a 1:1:1:2 chance of receiving Duloxetine. Those who are eligible to receive three of the four treatments have an equal chance of receiving one of the remaining 3 treatments. For second stage randomization, we assume 33% response across all first stage treatments and generate 16 randomization lists depending on initial treatment and eligibility for second stage treatment. For those patients who are eligible to receive Duloxetine (and did not receive it in the first stage), patients will be assigned by block randomization with 1:1:4 weights to Duloxetine. Otherwise, patients are equally assigned among treatments not initially received. Random block sizes depend on the size of the list.

#### Treatments under study and intervention procedures

The four treatments were selected because each has a unique mode of delivery, level of patient involvement (passive vs active involvement) and presumed mechanisms of action. Below is an overview of each treatment, the predictors of response to treatment and the procedures for delivery of each.

*Mindfulness-Based Stress Reduction (MBSR) overview.* Mindfulness-based interventions (MBIs) such as mindfulness-based stress reduction (MBSR) are widely used non-pharmacological interventions for pain reduction involving mindfulness meditation,^18–20^ and are now recommended in many treatment guidelines for cLBP.^21–23^ MBSR is typically delivered in a group setting with trained providers. Weekly sessions take place over 8 to 12 weeks and require extensive homework. Meta-analyses report that MBIs reduce pain intensity and pain interference in chronic pain syndromes, including cLBP with effect sizes of 0.3–0.5.^20^^*,*^^24–26^ MBIs also improve the depression, anxiety, and addiction^27–29^ that often accompany chronic pain, and have been found to lessen opioid misuse in people with chronic pain.^30–32^

*Predictors of response to MBSR using light phenotyping (Aim 2).* We have a priori hypotheses regarding the patient reported outcomes (PROs) that will identify a subset of cLBP patients who will preferentially respond to a pain-related MBI, in particular, psychological/emotional components of reactivity to pain that can exacerbate pain unpleasantness and interference. Since MBIs have shown efficacy for nociplastic, neuropathic pain, and nociceptive pain, we predict MBSR will show similar efficacy across these pain types. We predict that patients who preferentially respond to MBSR will have *higher baseline levels of pain catastrophizing,* as measured by the Pain Catastrophizing Scale (PCS),^33^ or *lower scores on the Experiences Questionnaire (EQ).*^34^

*Predictors of response to MBSR using deep phenotyping (Aim 3).* After a 4-week MBSR course, individuals with cLBP had significantly increased activity in the subgenual anterior cingulate cortex (sgACC) and ventrolateral PFC—two regions known to play a critical role in the descending inhibition of pain.^35^ These results are supported by a study of healthy volunteers conducted by Zeidan et al.^36^ that found increased activity in the sgACC, as well as orbitofrontal cortex and insula and decreased activity in the primary somatosensory cortex (S1) and thalamus during evoked pain stimulation following mindfulness training. These studies suggest MBSR acts in part by enhancing central inhibitory responses to pain. We therefore hypothesize that cLBP patients with *decreased activation in response to pain in the sgACC and PFC* and *increased activation in S1 and thalamus* at baseline will respond preferentially to MBSR.

*Mindfulness-based stress reduction procedures.* Participants randomized to MBSR will meet for 8 weekly 2-hour group sessions and one 6-hour “retreat” with a masters-level or higher therapist formally trained in MBSR and with experience working with chronic pain patients. MBSR for pain is manualized and includes all the components of standard MBSR.^19^^,^^37^^,^^38^ Each session includes practicing formal mindfulness exercises, dialogue and “mindful inquiry” with the therapist and group, and didactic information (eg, stress and pain physiology, using mindfulness for coping with stress and pain). Patients are asked to practice daily formal mindfulness at home using assigned audio recordings of 30–45-minute guided mindfulness exercises streamed from a study link to Qualtrics. For more information about MBSR and the other interventions, including the assessment of participant adherence and fidelity of treatment, please see Supplement 3 at https://medicine.umich.edu/dept/cpfrc/resources-0.

*Physical therapy and exercise overview.* Physical therapy (PT) and exercise are amongst the most recommended treatments for cLBP. PT consists of a variety of approaches such as manual therapy, directional preference exercises, and nerve mobilization procedures that are tailored to patients based on their movement characteristics. PT is typically delivered one-to-one, in person, and by trained physical therapists. PT is supplemented by exercise done outside of the clinic setting that can include aerobic exercise, stretching and walking.^39^

*Predictors of response to PT using light phenotyping (Aim 2).* Most studies to date that have attempted to identify the factors that are most predictive of differential responsiveness to exercise in cLBP have been based on some variation of the cognitive behavioral fear avoidance model, wherein low functional self-efficacy for exercise is related to high pain catastrophizing and fear of movement.^40–44^ This cognition has been shown to promote the transition from acute to cLBP, as well as to be associated with worse chronic low back pain.^44–46^ Our primary hypothesis is those individuals with *higher baseline levels of fear avoidance (higher scores on the Fear Avoidance Beliefs Questionnaire [FABQ]*^47^*)* and *lower levels of self-efficacy (lower scores on the PROMIS Self-Efficacy for Managing Symptoms questionnaire*^48^*)* will be most likely to improve from our PT program, which is focused on getting participants over this fear of movement.

*Predictors of response to PT using deep phenotyping (Aim 3).* Elevated basal inflammation (eg, CRP, IL-6) is associated with both the presence and severity of cLBP even after adjustment for potentially confounding variables like obesity.^49^^,^^50^ Exercise is known to exert anti-inflammatory effects and has been shown to decrease levels of inflammation substantially.^51^^,^^52^ Also, vagal tone is low in many chronic pain patients and related to the duration of time individuals have had pain^53^ and heart rate variability (HRV) has been shown to improve following even milder exercise programs such as the one we propose.^54–62^ We therefore anticipate that *high basal inflammation* and *low vagal tone at baseline* will predict responsiveness to the PT program.

*Physical therapy and exercise procedures.* Participants randomized to PT will meet with the physical therapist twice a week for a 1-hour session for weeks 1 and 2 and then weekly for the remaining 6 weeks. After taking a thorough history, an examination is performed, then the physical therapist will tailor a program to the participant’s needs according to recommended PT practice guidelines that will include in-person treatment, home exercise prescription, and encouragement of progressive, low-intensity, submaximal fitness and endurance activities, such as walking.^39^^,^^63^ Participants will be given a home program of exercises to be done daily and asked to engage in daily walking with a set goal based on the individual’s capacity and current fitness level. Based upon the progress, the physical therapist will make any necessary modifications to treatment.

*Acupressure mHealth self-management overview.* Acupuncture is a component of traditional Chinese medicine (TCM), and research over the past three decades has shown that acupuncture is effective for the treatment of chronic pain.^64^ Acupressure is a related technique wherein pressure is applied via a finger or device to specific acupoints. Acupressure is highly scalable and can be taught to patients (for self-administration) and supported using technology. While less research has been performed on self-administered acupressure, emerging data indicates that self-acupressure is effective for chronic pain^65^^*,*^^66^ and low back pain specifically.^67–70^ In our own studies, the mHealth app used here resulted in significant improvements in pain, fatigue, sleep, and depression for 288 fatigued breast cancer survivors^71^^*,*^^72^ and reduced low back pain compared to usual care (35% reduction, *P* < .05) in cLBP.^73^

*Prediction of response to acupressure using light phenotyping (Aim 2)*. The literature regarding prediction of acupressure effects is minimal. However, as mentioned above, these therapies are thought to work primarily via central nervous system mechanisms. As such, they should be more effective in addressing nociplastic pain. Although no groups that we are aware of have looked directly at this issue, our group has preliminary unpublished data in cLBP patients treated with acupuncture (n = 19; treated 6 times over a 4-week period with pain assessed prior to and immediately after each treatment, and widespread pain assessed by the number of body regions having pain) showing a significant relationship between increased baseline widespread pain and subsequent acupuncture response (Standardized Beta [adjusting for age and sex] = 0.58, t = 2.1, *P* = .048). These pilot data suggest that nociplastic pain may be an important marker of acupuncture treatment outcome. In further support of this hypothesis, Witt et al. noted that females were more likely to respond to acupuncture than males, a phenomenon that is noted when treatments work primarily in the CNS, as with duloxetine.^74^^,^^75^ As such, we predict that *females* with cLBP will respond better to acupressure than men, as will those with greater nociplastic pain as indicated by higher scores on the *2016 Fibromyalgia Survey Questionnaire (FSQ)*.^76^

*Prediction of response to acupressure using deep phenotyping (Aim 3).* We are aware of no studies to date that have examined the predictive ability of QST or our other deep phenotyping methods in determining pain improvement following self-administered acupressure. That said, in the context of acupuncture, we were one of the first to show that pressure pain thresholds at baseline were differentially predictive of verum (active) and sham acupuncture.^77^^,^^78^ Patients who had higher pain thresholds were more likely to respond to verum acupuncture. We interpret this result to mean that patients with less *nociceptive* pain sensitivity respond better to acupuncture needling. We predict that cLBP patients with *higher pain thresholds* on QST will also respond better to acupressure. There is a strong relationship between sensory cortex brain activity and acupuncture response, as the primary somatosensory cortex (S1) has been shown to be involved in acupuncture effects in fibromyalgia and carpal tunnel pain.^79^ Further, we found a significant correlation between the reduction in posterior insula glutamate and chronic pain in nociplastic pain patients following acupuncture.^80^ We also found similar relationships between insula to DMN connectivity wherein reductions in this connectivity were correlated with improvements in clinical pain following acupuncture in this population.^81^ As such, we predict that cLBP patients with *higher posterior insula glutamate* and/or *greater insula—DMN connectivity,* as well as *increased DMN-S1 connectivity* at baseline will display an improved analgesic response to self-administered acupressure.

*Acupressure self-management procedures*. The self-administered acupressure intervention will be delivered using the modified MeTime Acupressure mobile app in addition to in-person instruction via study staff. Participants will also receive a hand-held pressure monitor and manual stimulation tool (referred to as an AcuWand; Arbor Medical Innovations, LLC) to be used in association with the acupressure app to help participants apply the correct amount of pressure to acupoints. Study participants will be told to perform acupressure once per day and to stimulate each point a circular motion for 3 minutes. There are 9 acupressure points, totaling 27 minutes of stimulation per day.

*Non-opioid pharmacotherapy (duloxetine) overview.* Duloxetine is a serotonin norepinephrine reuptake inhibitor (SNRI) that is FDA-approved for use in cLBP,^82–84^ and, as such, is included as a recommended therapy in nearly all current treatment guidelines for low back pain. Hence, duloxetine is a logical non-opioid analgesic to include in our SMART trial. Duloxetine and other drugs that increase both serotonergic and noradrenergic activity (eg, tricyclics) are thought to work as analgesics by increasing activity in descending anti-nociceptive pathways.^85^

*Predictors of response to duloxetine using light phenotyping (Aim 2).* We have several a priori hypotheses regarding the PROs that will identify a subset of cLBP patients who will preferentially respond to duloxetine. We hypothesize that we will replicate previous studies suggesting that cLBP participants will preferentially respond to this therapy if PROs indicate stronger elements of either neuropathic pain (indicated by a high PainDETECT score^86^^,^^87^) or nociplastic pain (indicated by higher scores on the FSQ^75^).

*Predictors of response to duloxetine using deep phenotyping (Aim 3).* We and others have also performed QST and/or neuroimaging studies that suggest that the subgroup of cLBP patients with either neuropathic or centralized/nociplastic pain will preferentially respond to SNRIs. Yarnitsky et al. showed that the subset of neuropathic pain patients with diminished endogenous pain inhibition, measured using a conditioned pain modulation (CPM) procedure,^88^ were more likely to respond to duloxetine. Our group has performed a series of studies with a different SNRI, milnacipran, and showed that the drug preferentially works in individuals with a brain imaging pattern consistent with decreased descending analgesia, namely, decreased connectivity between the periaqueductal gray (PAG) and the insular cortex, as well as between the rostral part of the anterior cingulate cortex and the insular cortex.^89^ We have shown that the stimulated inflammatory response (ie, inflammation after LPS-stimulation) is strongly associated with nociplastic pain characteristics such as multifocal pain and the number of pain syndromes present in the MAPP study.^90^^,^^91^ We anticipate then that deficient pain inhibition on QST, decreased PAG-insula connectivity, and elevated stimulated inflammatory responses at baseline will be associated with a positive response to centrally acting duloxetine.

*Duloxetine procedures.* Participants randomized to the duloxetine arm will review the dosing schedule and safety information for the medication at the pre-intervention visit (T2 for Treatment 1 or T3 for Treatment 2) with the study coordinator. Findings from the physical exam conducted at baseline, as well as potential drug contraindications will be reviewed as an additional precautionary measure. Participants will then be given 105 pills of 30 mg duloxetine with an 8-week dose escalation schedule and an additional 11 pills for those who would like to taper. Participants will be asked to start taking the medication from home, 7 days after the pre-intervention visit (T2/T3 visit). During the entire 8-week intervention, patients will be asked to keep a daily log of medication dosage, any missed doses, and any side-effects they may have experienced.

### Retention and subject incentives

The study team’s priority is to facilitate and support participation in the study (ie, lessen participant burden). When possible, research appointments will be scheduled on the same day as standard care appointments. Research appointments are scheduled through the UMH electronic medical record system known as “MiChart,” and thus appointment reminders are automatically sent via text and an automated call system prior to the appointment. Most data collection visits will use a hybrid approach where questionnaire data will be collected online, while clinical data such as vitals, biospecimen collection, and functional testing will take place on site. For study integrity, participants with missed visits will be contacted 3 times to reschedule. Attempts will be made again to follow up at the next study visit window. If there is no contact with the participant for 9 months, they will be reported as lost to follow-up. Subjects will receive incentive payments upon completion of each study visit and can receive up to $550 for light phenotyping and up to an additional $500 for deep phenotyping.

### Trial oversight and procedures for recording and reporting serious adverse events

This study has oversight by a Data and Safety Monitoring Board (DSMB) that acts in an independent, advisory capacity to the study sponsor, National Institute of Arthritis and Musculoskeletal and Skin Diseases (NIAMS), to monitor study progress, data quality, and accumulation of safety data, to alert the Institute regarding any potential safety or other monitoring concerns affecting study conduct. The DSMB has access to the study protocol, consent forms, and other pertinent study related documents, in addition to comprehensive reports with study data to aid in the data and safety monitoring for study duration. The DSMB will meet at least semiannually to assess safety and efficacy data from each arm of the study.

A serious adverse event (SAE) is defined as any adverse event that results in one or more of the following outcomes: death, a life-threatening event, inpatient hospitalization, or prolongation of existing hospitalization relating to study treatment, a persistent or significant disability/incapacity hospitalization relating to study treatment, or an important medical event based upon appropriate medical judgment. SAEs that are related to a study intervention are reported to the NIAMS Executive Secretary who will report to the DSMB and NIAMS within 48 hours of the study team becoming aware of the event. The UM IRBMED is notified of the SAE within 7 days of occurrence. SAEs that are unrelated to the study interventions are also reported within 48 hours but are reported to the UM IRBMED in the annual report prior to scheduled continuing review.

### Data management

All data collected on study participants will be obtained and managed specifically for research purposes. The types of data to be collected in aggregate across projects include medical status and history; self-report questionnaires that assess physical and psychological symptoms and life functioning; physical exams; functional performance measures; participant responses to all QST and physiological performance measures; biospecimens; and neuroimaging data (^1^H-MRS, fMRI, functional connectivity MRI). Imaging data will be obtained using one of two 3.0 T GE MRI scanners located at the Functional Magnetic Resonance Imaging lab at University of Michigan or at the University Hospital 3.0 T Philips scanner. Participants will also be asked to provide blood samples. All blood samples will be de-identified prior to storage. All samples will be collected, securely stored, and processed for disbursement and analysis by appropriate study investigators. Participant identity and confidentiality will be maintained throughout.

### Statistical design and analysis

We focus on predicting differential analgesic responses to treatments and other secondary endpoints and develop a tool to predict treatment response and gain exploratory structural insights into the causal relationships between light and deep phenotyping measures. Hypotheses to be tested include improved responses to specific treatments for groups of patients defined by covariates, including the ones measured at light and deep phenotyping. In addition to sex and other key demographic and clinical variables, this includes groups of patients defined by higher pain catastrophizing; psychological/emotional components of reactivity to pain; increased activity in the subgenual ACC and ventrolateral PFC brain regions; higher scores on the FABQ^47^ and lower scores for PROMIS measures;^48^ low vagal tone and high basal inflammation; brain neurotransmitters; co-occurring sleep dysfunction; and higher scores on the FSQ.^76^ Deep phenotyping includes QST, fMRI, and measures of inflammation and ANS function to identify key neurobiological markers of cLBP.

Descriptive and univariate model-based analyses will be used to guide an initial approach to data analysis and multivariate predictive model trimming. These analyses will include among other things assessment of data quality, relationships between phenotype features prior to each treatment, correlations between phenotype variables and responses, patterns of missing data, univariate model-based analyses. The adequacy of proposed models with be assessed by model diagnostic plots and tests.

Multivariate longitudinal data analysis will be the primary analytic tool. Hypothesis testing will be model based using likelihood ratio tests. The main hypotheses of differential treatment effects (treatment moderators, treatment effect modifiers) will be handled by introduction of the interaction terms between the phenotype and treatment indicator variables. Dependent on the scale of the response variables, we use multivariate linear, logistic binary, and ordinal mixed models. Gaussian subject-specific intercept term will be used to model the effect of unmeasured factors shared by longitudinal observations on the same subject. Coarsening of the continuous response variables to ordinal or binary will be considered for robustness and clinical value as well as diagnostics of the main-line continuous models. We select best models using the unbiased Bayesian Information Criterion (BIC). We also use 10-fold cross-validation to protect against overfitting the model and assess the model’s predictive performance. To deal with potentially high-dimensional predictive features, regularized Elastic Nets regression penalties will be considered. Penalties in the likelihood function will follow the Elastic Nets family with LASSO favored for its feature elimination potential. Machine-learning model-free algorithms (random forests, SVMs) will be utilized to explore predictions. In exploratory analyses, we examine changes in neurobiological markers following treatment. These analyses help us determine how these treatments uniquely affect pain mechanisms, a critical step for the development of new analgesics.

*Causal analysis*. We conduct exploratory analysis of causal relationships between light and deep phenotypic factors and analgesic and secondary response variables to assess neurobiological and inflammatory biomarkers (deep phenotyping variables) as potential mechanistic mediators of the treatment effects of light phenotypic measures. The results will be expressed in terms of the proportion of the treatment effect (PTE) explained by the biomarker with bootstrap used to obtain standard errors and the Wald test for the presence of the mediation effect. Another application of causal models (counterfactual causal inference) will be used to disentangle the learning effect of the patient history that contributed to the treatment decision from the benefit of treatment to the patient with the specific history.

*Missing data*. Handling of missing data will include descriptive analysis of missing data patterns followed by the analysis of reasons for missingness as a nominal response using multinomial logistic regression. Sensitivity analyses will include missing data imputation by predictive-matching algorithms and missing data exclusion under a missing-at-random assumption. Multiple imputation approaches will be utilized if the fraction of missing data is substantial (more than 15%).

*Power*. The power for correlating within-patient improvement due to one of the four main treatments with a phenotypic variable will reach 86% for correlations of 0.3 or higher, conservatively assuming the model is applied to one randomized treatment segment with 100 patients. This corresponds to first phase or second phase treatment in one of the four treatment groups, and a model based on the light phenotyping patient group of 400 total. Assessing the power for prediction, we expect that at least 30% of patients will show 50% improvements in analgesic outcomes under each of the treatments. We expect the predictive panel of light phenotypic variables to show AUC exceeding a clinically relevant AUC of 0.7, dependent on the specific setting. Under this assumption we will have the power of at least 91% to reject the null hypothesis AUC of 0.5 by a two-sided test in the subgroup analysis setting described above.

### Dissemination of results

This study will be conducted in accordance with the National Institutes of Health (NIH) Public Access Policy, which ensures that the public has access to the published results of NIH funded research. It requires scientists to submit final peer-reviewed journal manuscripts that arise from NIH funds to the digital archive PubMed Central upon acceptance for publication.

#### Expected outcomes and future directions

Evidence-based treatments for chronic low back pain typically work well in only a fraction of patients, and at present there is little guidance regarding what treatment should be used in which patients. Our central hypothesis is that *an interventional response phenotyping study can identify individuals with different underlying mechanisms for their pain who thus respond differentially to evidence-based treatments for cLBP.* Thus, using a SMART design study, we expect to identify the subsets of participants that respond to each of four commonly used treatments, physical therapy, a behavioral group therapy, mHealth self-management and medication. Each treatment requires different levels of patient engagement and is presumed to work via different mechanisms. As such, we will first identify which currently available, clinically derived measures can predict differential responsiveness to the various treatments. Next, we will identify experimental measures such as neuroimaging, QST, inflammatory markers, autonomic tone, biomechanical metrics, and others that predict differential responsiveness to the treatments, as well as to infer mechanisms of action.

This research will address a critical need, which is to attain high quality information on individuals cLBP that can predict which non-pharmacological, pharmacological, or procedural therapies work best for chronic low back pain patients. This work represents an important step forward in reaching the ultimate goal of realizing personalized medicine for people with cLBP. These data can serve as a launching point to inform for future studies that also aspire for the objectives of the larger BACPAC vision—the development of algorithms that will advise clinicians regarding the best treatment approach for each unique patient that they encounter.

## Supplementary Material

pnad005_Supplementary_Data
