# Supplementary material for: An Interventional Response Phenotyping Study in Chronic Low Back Pain: Protocol for a Mechanistic Randomized Controlled Trial
Source: Pain Med. 2023 Jan 27;24(Suppl 1):S126–38. doi: 10.1093/pm/pnad005 (PMC10403311; doi:10.1093/pm/pnad005)
Supplement: pnad005_Supplementary_Data [file pnad005_supplementary_data.zip › pnad005_Supplementary_Data/UM SMART BACPAC Suppl 3 Tx Activities Adherence and Fidelity.pdf]

Supplement 3. Treatment Activities, Adherence, and Fidelity

Schedule of Activities for the MBSR intervention

Session	Intervention	Outcomes measured
1	Introducing Mindfulness Meditation: Doing to Being	
2	Perception & Knowing Are Different	Chronic Pain Acceptance Questionnaire (CPAQr-8) & Experiences Questionnaire (EQ-4)
3	There is Pleasure & Power in Being Present	
4	Stress Reactivity	Chronic Pain Acceptance Questionnaire (CPAQr-8) & Experiences Questionnaire (EQ-4)
5	Stress, Mindful Awareness & Responding	
6	Stressful Communications	Chronic Pain Acceptance Questionnaire (CPAQr-8) & Experiences Questionnaire (EQ-4)
7	How Do I Best Take Care of Myself?	
8	Endings Are Beginnings	
4-hour mini retreat	Occurs between weeks 5-7	

Participant Adherence- MBSR

Participants will also be asked to keep a log of study related activities completed weekly via online surveys (Qualtrics). Attendance will be taken at each MBSR session. Patient homework adherence can be estimated by download count of each mp3 on streaming site.

Standardizing Assessment and Intervention- MBSR

Fidelity will be supported by use of a treatment manual. Delivery of the intervention will be facilitated by in-session therapist checklists. All sessions will be video recorded, and 20% will be observed and scored for adherence and fidelity.[76] Weekly provider supervision and fidelity check meetings are held.

Schedule of Activities for the Physical Therapy & Exercise Intervention

After taking a thorough history, an examination is performed. This examination includes self-reported pain severity and disability as well as clinical assessment such as valuation of strength/endurance, neural mobility, range of motion of the spine and hips, mobility of vertebral segments, and movement patterns that are difficult or avoided (i.e., directional preference). The physical therapist will tailor a program to the participant's needs according to recommended PT practice guidelines that will include in-person treatment, home exercise prescription, and encouragement of progressive, low-intensity, submaximal fitness and endurance activities, such as walking.[44,77] The in-person treatment focuses on three main areas: mobilization (i.e., gaining joint mobility), flexibility (soft tissue and neural mobility), and strengthening (see supporting documents for PT Treatment Manual). Manual therapy is a main treatment component, and exercises include repeated directional preference movements, trunk flexion and stabilization. Exercises address both specific deficiencies noted in evaluation, as well as coordination of body motions. Participants will be given a home program of exercises to be done daily and asked to engage in daily walking with a set goal based on the individual's capacity and current fitness level. Walking was selected as the aerobic exercise of focus for this treatment because it is recommended for patients with all levels of pain severity, is highly feasible to complete, and has shown effects on outcomes such as pain and disability.[78,79] At weekly visits with the physical therapist, participants will discuss progress toward meeting their walking goal and adherence to the home exercise program. Based upon the progress, the PT will make any necessary modifications to treatment.

Sessions (week)	Aerobic Warm Up	Manual Therapy	Mobility	Questionnaires
1&2 (week 1)	5 minutes	None	Directional preference	Oswestry Disability Index (ODI) Numeric Pain Rating Scale (NPRS)
3&4 (week 2)	5 minutes	No more than 15 minutes	Continue directional preference and add hip stretches as indicated	Numeric Pain Rating Scale (NPRS)
5 (week 3)	5 minutes	No more than 15 minutes	Same as previous visit	Numeric Pain Rating Scale (NPRS)
6 (week 4)	5 minutes	No more than 15 minutes	Same as previous visit	Numeric Pain Rating Scale (NPRS)
7 (week 5)	5 minutes	No more than 15 minutes	Same as previous visit	Numeric Pain Rating Scale (NPRS)
8 (week 6)	5 minutes	No more than 15 minutes	Same as previous visit	Numeric Pain Rating Scale (NPRS), Patient global impression of change (PGIC) Oswestry Disability Index (ODI)
9 (week 7)	5 minutes	As indicated	No more than 5 min	Numeric Pain Rating Scale (NPRS)
10 (week 8)	5 minutes	As indicated	No more than 5 min	Numeric Pain Rating Scale (NPRS), Patient global impression of change (PGIC) and Oswestry Disability Index (ODI)

Participant Adherence- PT and Exercise

Participants will be asked to keep a log of the home exercises they are able to complete and the time spent on each activity. Attendance for the scheduled PT sessions will also be recorded.

Standardizing Assessment and Intervention- Physical Therapy and Exercise

Standardizing Examination: Baseline examination will be performed by a physical therapist investigative team member. These assessors will go through a two-hour training session to standardize examination procedures. During training, special tests will be reviewed *to* ensure correct performance of measures. Assessors will also review inclusion and exclusion criteria. A checklist will be provided in the standardized examination template to ensure that participants meet eligibility requirements. Fidelity checks will be completed on a semi-annual basis.

Intervention Training: Study faculty and residents currently enrolled in or recently graduated from the Michigan Medicine - University of Michigan Orthopedic Residency Program will provide all intervention. Within the curriculum, residents receive detailed, standardized instruction on research methods and intervention techniques. Residents who will be providing intervention will undergo an additional two-hour training and follow the BACPAC PT training manual (see supporting documents). The program director, Laura Fisher, will provide coverage of care as needed and oversee intervention training. All residents will receive PEERRS and CITI training and be added to the IRB.

Schedule of Activities for the Acupressure Intervention

The self-acupressure intervention will be delivered using the modified MeTime Acupressure mobile app in addition to in-person instruction via study staff. The MeTime Acupressure app was developed in association with patient focus groups (six focus groups each of eight to ten women) and the University of Michigan 3D Media Laboratory (screen shot examples in 1). The MeTime Acupressure app will be loaded onto computer tablets or smart phones by the participants. Participants will also receive an AcuWand to be used in association with the acupressure app to help participants apply the correct amount of pressure to acupoints (See Figure 2). The self-acupressure intervention points will consist of *Du 20*, Conception Vessel 6 (CV- 6), Large Intestine 4 (LI-4), Stomach 36 (ST-36), Spleen 6 (SP-6), and Kidney 3 (K-3) (See 3). Points will be administered bilaterally except for *Du 20*, and CV-6, which were done centrally.

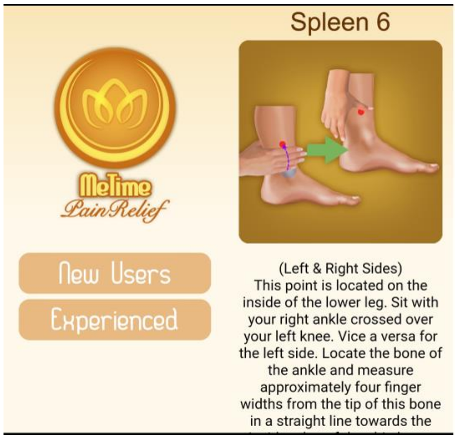

Figure 1 Sample MeTime Acupressure App

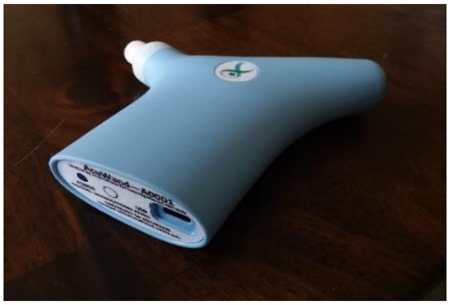

Figure 2 AcuWand Device

Participants will be introduced to the MeTime App and AcuWand device at their clinic visit. A research team member will provide the patient with instructions and guide the patient through using the app, the AcuWand and performing Self-Acupressure. Study participants will be told to perform acupressure once per day and to stimulate each point in a circular motion for three minutes. There are 10 acupressure points (Figure 3), totaling 30 minutes of stimulation per day.

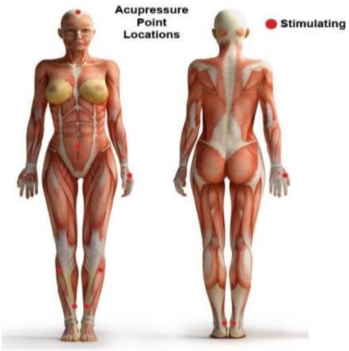

Figure 3 Location of Acupressure Points

Participant Adherence- Acupressure

Participants will be asked to log time spent using the AcuWand daily and record any reasons for missed sessions. The Acuwand device also records the time a participant spends using the device. A check in call will be made by the study team 48 hours after starting the treatment to address any patient questions or concerns. An additional call with be made two weeks after start of treatment to assess adherence. Text messages remind participants to charge the AcuWand device will be sent at week 4 and 6 of treatment.

Standardizing Assessment and Intervention

Fidelity of the acupressure intervention in study staff, who are teaching participants acupressure, i.e., acupressure educators will be assessed by study investigator Rick Harris every six months. Educators will be trained using the BACPAC Acupressure Therapist Treatment Manual.

Schedule of Activities for the Duloxetine Intervention

Participants randomized to the duloxetine arm will review the dosing schedule for the medication and safety information for the medication at the pre-intervention visit (T2 for Treatment 1 or T3 for Treatment 2) with the study coordinator. At the baseline visit a physical exam was performed by a physician and drug contraindications will be reviewed as an additional precautionary measure.

Participants will then be given 105 pills of 30mg duloxetine with an 8-week dose escalation schedule and an additional 11 pills will be provided for those who would like to taper. Participants will be asked to start taking the medication from home, 7-14 days after the pre-intervention visit (T2/T3 visit). Participants will be mailed the study medication by the research pharmacy, arriving 1 day prior to the start date of the medication.

The first day of the medication will be day 1 and subjects will be scheduled for a phone visit with the study team at day 7 and day 42 (or the next business day if this falls on a weekend). For days 1-7, patients will take 30mg of duloxetine once a day, in the morning. Starting day 8, participants tolerating the medication will be escalated to 60mg once a day. They will also have the option of staying at 30mg once a day or stopping the medication (see Figure 4 below).

Participants will be scheduled for a phone visit with the study team at the end of week 1 to discuss dose escalation and at the end of week 6 to discuss dose taper. At the end of the 8-week intervention period, participants will have the option to continue the medication

commercially (non-study medication) under the care of their physician or taper off the medication. During the entire 8-week intervention, patients will be asked to keep a daily log of medication dosage, any missed doses, and any side-effects they may have experienced.

Dose Escalation

At the day 7 call, the study staff will document any adverse events and safety concerns the patients may have. Willing subjects will be asked to increase the dose to 60mg, once a day, in the mornings. Participants will also have the option of staying at a dose of 30mg per day or stopping the medication entirely. If day 7 is on a weekend, the call will be scheduled for the next business day.

Dose Taper

At the day 42 call, the study team will document any adverse events and safety concerns. Participants who are tolerating duloxetine and want to continue therapy using commercial medication, will be asked to obtain a prescription for duloxetine from their primary care physician, or other provider. The study team can provide a letter with drug information. If at this time the patient is not interested in continuing duloxetine, the taper schedule will be explained (see Figure 4 below).

- A patient at the max dosage of 60mg will be asked to taper down starting day 56 to 30mg for 7 days and then 30mg every other day for another 7 days, before stopping the medication completely. These additional 11 pills will be shipped to the patient and arrive on day 56 of the treatment. If day 42 is on a weekend, the call will be scheduled for the next business day.
- A patient on 30mg, will be asked to start tapering on day 56 by taking 30mg every other day for another 7 days, before stopping the medication completely.

Duloxetine Post-Intervention Visit

When patients arrive for their post-intervention visit, the study team will document any adverse events, and/or safety concerns the participants may have. A daily log of medication dosage will also be collected from the patient at this point. Unused pills will be counted, documented, and will be returned to the research pharmacy for accountability. Any additional pills for tapering will be provided for participants who want to taper.

Medication Procurement and Storage

Medications will be mailed from the research pharmacy to participants as needed. If pickup by study staff is required, duloxetine will be stored in the research room's double locked cabinet. Temperature in the room must be managed between 20 -25°c (68-77°F); excursions permitted to 15-30°C (59-86°F) – per package insert. A log of maximum/minimum readings will be entered into a log while the drug is in research storage.

Medication Accountability

All study medication bottles will be collected/returned from the participant to the study team. When a bottle is returned, a study team member will count how many capsules or left and record the number on the bottle, date, and initials. All medication bottles will be returned to the Research Pharmacy for drug accountability. The Research Pharmacy will be asked to sign off on the accountability log showing the bottle is returned. The Research Pharmacy will dispose of medication and bottles.

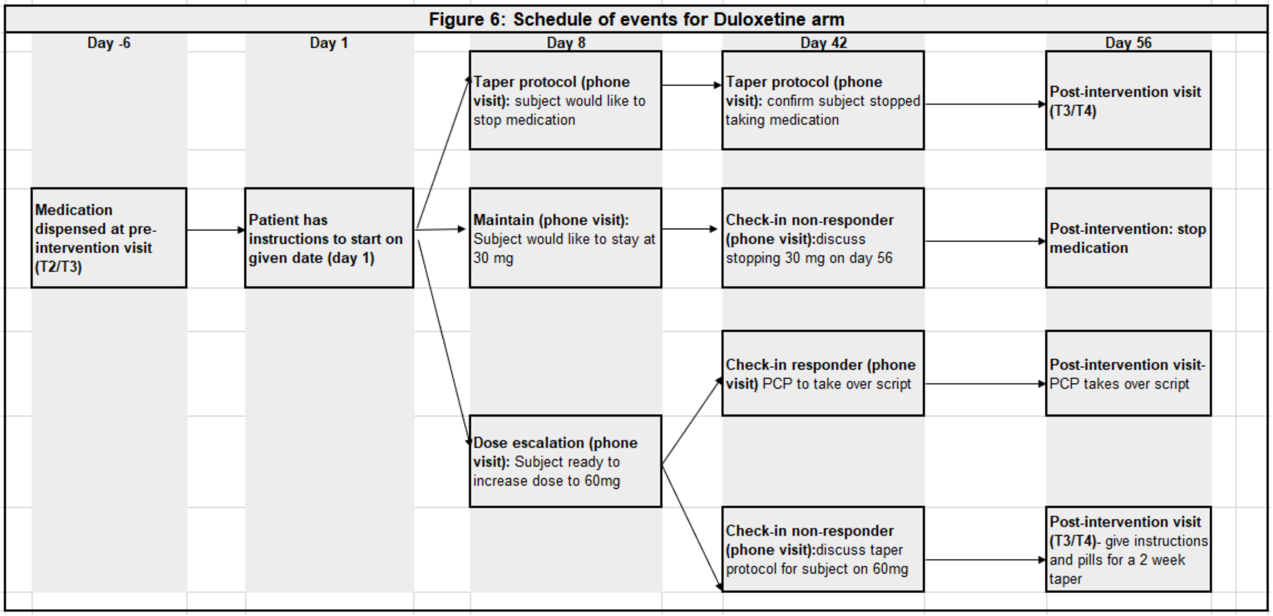

Figure 4: Schedule of Events for Duloxetine Arm
