# Supplementary material for: An Interventional Response Phenotyping Study in Chronic Low Back Pain: Protocol for a Mechanistic Randomized Controlled Trial
Source: Pain Med. 2023 Jan 27;24(Suppl 1):S126–38. doi: 10.1093/pm/pnad005 (PMC10403311; doi:10.1093/pm/pnad005)
Supplement: pnad005_Supplementary_Data [file pnad005_supplementary_data.zip › pnad005_Supplementary_Data/UM SMART BACPAC Supplement 1 Schedule of Activities and Data Collected.pdf]

Supplement 1. Schedule of Activities and Associated Data Collection

	Scheduled																		Unscheduled Visits	
	Pre-screening (-30days)	Study Visit T1 (Week 1)	Ecological Momentary Activity (EMA)-1 (Week 1)	Study Visit T2 (Week 6)	Deep Visit 1 ≠ (Week 6)	Ecological Momentary Activity (EMA)-2 (Week 6)	Intervention 1 (Weeks 7-14)	Study Visit T3 (Week 15)	Deep Visit 2 ≠ (Week 15)	Ecological Momentary Activity (EMA)-3 (Week 15)	Intervention 2 (Weeks 116-23)	Study Visit T4 (Week 224)	Ecological Momentary Activity (EMA)-4 (Week 24)	Ecological Momentary Activity (EMA)-5 (Week 36)	Study Visit T5 (Week 36)	10 Mini-assessments (Weeks 3, 8, 10, 17, 21, 26, 28, 30, 32, 34)	1 Expanded Mini-assessment (Week 12)	1 Mediation Mini-assessment (Week 19)	Unscheduled Non-study Intervention Triggering Deep	Other Non-Study Intervention Triggering Follow-up Post Treatment (4&8 weeks post
EMR Eligibility Review	x																			
Screening Eligibility Review	x	x																		
Informed Consent		x																		
Charlson Comorbidity Index (CCI)		x																		
Demographics		x																		
Medical History and Medications		x		x				X				x			X					
Vitals, Height & Weight		x		x				X				x			X					
**Biomechanical, Physical Exam & Physical Function Measures**																				
2-Minute Walk Test (2MWT)<br>*4-Meter Walk Gait Speed Test (from SPPB)*<br>*Timed Sit-to-Stand (STS)*		x		x				X				x			X					
Neurotesting-Patellar Deep Tendon Reflex		x					b				b									

Neurotesting-Achilles Deep Tendon Reflex		x					b				b									
Neurotesting-Sensation		x					b				b									
Neurotesting-Ankle Dorsiflexion (L4)		x					b				b									
Neurotesting-Great toe extension (L5)		x					b				b									
Neurotesting-Hamstrings (S1/S2)		x					b				b									
Neurotesting- Single Leg Calf Raises (S1/S2)		x					b				b									
Neural Tension- Passive Straight Leg Raise + Tightness		x					b				b									
Cross Straight Leg Raise		x					b				b									
SI Provocation- Compression		x					b				b									
SI Provocation- FABER Test		x					b				b									
Fortin Test		x																		
Neurotesting-Hip flexion (L2/L3)							b				b									
Neurotesting-Quadriceps (L3/L4)							b				b									
Neurotesting-Seated Slump Test with Active Straight Leg Raise (ASLR)							b				b									
Neurotesting- Beighton Score (Pinky, Thumb & Elbow)							b				b									
Neurotesting- Beighton Score (Knees & Hands to floor)							b				b									

Hip Abduction-Dynamometer							b				b									
SI Provocation-Distraction							b				b									
SI Provocation-Thigh Thrust							b				b									
SI Provocation-Gaenslen's							b				b									
SI Joint- Active Straight Leg Raise							b				b									
SI Provocation-Sacral Thrust							b				b									
Hip Extension Dynamometer							b				b									
Lumbar Segmental Mobility (PA Spring Test)							b				b									
Prone Instability Test							b				b									
Inclinometer- Flexibility-Flexion, Extension and Side bending							b				b									
Observation for Aberrant Motion							b				b									
Directional Preference-Repeat flexion, extension, side glide							b				b									
Hip Provocation- FADDIR							b				b									
Pain Provocation-Quadrant Test							b				b									
Conity Device- Motion Assessments & Spine Kinematics Data		x																		
**Biospecimen Collection**																				

Whole blood<br>*Blood Serum*<br>*RNA PAXgene*<br>*Saliva*		x																		
Urine Pregnancy Test		x		x	x		x		x		x	x							x	
Imaging- MRI of Back and Pelvis		x																		
**Questionnaire Data**																				
Pain Duration and Frequency (cLBP)- 2 Items from NIH Research Task Force Minimum Dataset*		x			x				x								x			
Pain location- Radicular Pain Questions Adapted from NIH Research Task Force Minimum Dataset*		x			x				x								x			
Pain Somatization- Abbreviated Pain Somatization Adapted from NIH Research Task Force Minimum Dataset*		x			x				x								x			
Low Back Pain-Specific Pain Intensity*		x		x	x			X	x			x			X	x	x	x	x	x
Opioid use- Single-Item Current Opioid Use*		x		x	x			X	x			x			X		x			
Pain Intensity (PEG)*		x			x				x			x			x		x			
PROMIS Physical Functioning 6b*		x		x	x			X	x			x			X	x	x	x		
PROMIS Anxiety 4a*		x		x	x			X	x			x			X	x	x	x		
PROMIS Depression-4		x		x	x			X	x			x			X	x	x	x		

PROMIS Fatigue		x		x	x			X	x			x			X	x	x	x		
PROMIS Sleep Disturbance 6a*		x		x	x			X	x			x			X	x	x	x		
Sleep Duration*		x		x	x			X	x			x			X	x	x	x		
PROMIS Social Role Activity		x		x				X				x			X	x	x	x		
PROMIS Pain Interference 4a*	x	x		x	x			X	x			x			X	x	x	x	x	x
PROMIS Cognitive Function		x		x	x			X	x			x			X	x	x	x		
PROMIS Pain intensity		x		x	x			X	x			x			X	x	x	x	x	x
Pain Catastrophizing Scale (PCS)*		x		x	x			X	x			x			X		x			
Patient Health Questionnaire-2 (PHQ-2)*		x			x				x								x			
Generalized Anxiety Disorder-2 (GAD-2)*		x			x				x								x			
Patient global impression of change (PGIC)/Global Rating of change (GROC)*				x	x		b	X	x		b	x			X		x		x	x
Tobacco, Alcohol, Prescription medication, and other Substance use (TAPS)*		x			x				x								x			
Fibromyalgia (FM) Survey Criteria 2016 (Widespread Pain Index (WPI) and Symptom Severity Index (SSI))		x		X				X				x			X	x		x		
Widespread Pain*		x			x				x								x			

Life Orientation Test-Revised (LOT-R)		x		x					x				x		X					
Chronic Overlapping Pain Conditions Screener (COPCS)		x			x				x											
Pain Self-efficacy Questionnaire (PSEQ)		x		x				X				x			X					
PainDETECT		x		x	X			X	x			x			X					
Oswestry Disability Scale (ODI)		x		x			b	X			b	x			X					
Chronic Pain Acceptance Questionnaire (CPAQ-8)		x		x			c	X			c	x			X					
Fear Avoidance Beliefs Questionnaire (FABQ+Physical Activity)		x		x				X				x			X		x	x		
Experiences Questionnaire (EQ)-11		x		x				X				x			X		x	x		
Experiences Questionnaire (EQ)-5							c				c									
Perceived Stress Scale (PSS)		x		x				X				x			X					
Positive and Negative Affect Scale (PANAS)		x		x				X				x			X					
Childhood and Recent Traumatic Events Scale (CTES)		x																		
Credibility and Expectancy Questionnaire (CEQ)				x				X				x			X					
Client Satisfaction Questionnaire (CSQ)		x		x				X				x			X					

HEAL Treatment Expectancy v1.0 Short Form 6a		x		x				X				x			X					
Numeric Pain Rating Scale (NPRS)		x	x		x	x	b		x	x	b		x	x						
General Sensory Sensitivity (GSS-8)		x		x	x			X	x			x			X	x	x	x		
Ecological Momentary Activity (EMA) Mental and Physical Symptoms			x			x				x			x	x						
Low-Back Pain Treatment Categories Questionnaire		x		x				X				x					x	x		
Patient Preference Questionnaire		x																		
Patient Outcome Questionnaire		x																		
BACPAC Customer Service Questionnaire								x							x					
Randomization				a				A												
Adverse Event Reporting		x	x	x	x	x	x	X	x	x	x	X	x	x	X	x	x	x	x	x
**Deep Phenotyping Participants (n=160)**																				
Biospecimen					x				x										x	
RNA PAXgene																				
Blood Serum																				
Whole Blood																				
Neuroimaging- fMRI					x				x										x	
Conditioned Pain Modulation (CPM) Test					x				x										x	
Cuff Ascending and Pseudorandom					x				x										x	

Multimodal Automated Sensory Test (MAST) and Cuff Familiarization					x				x										x	
Multimodal Automated Sensory Test (MAST) Ascending					x				x										x	
Temporal Summation					x				x										x	
Tonic Cuff					x				x										x	
Two Point Discrimination Test (TPDT)					x				x										x	
Visual task					x				x										x	

≠ Only completed for deep phenotyping (n=160) participants

** Part of the HEAL minimum data set*

a Randomization will only occur if PGIC≥2

b Occurs if intervention is Physical Therapy

c Occurs if intervention is MBSR
