# Supplementary material for: An Interventional Response Phenotyping Study in Chronic Low Back Pain: Protocol for a Mechanistic Randomized Controlled Trial
Source: Pain Med. 2023 Jan 27;24(Suppl 1):S126–38. doi: 10.1093/pm/pnad005 (PMC10403311; doi:10.1093/pm/pnad005)
Supplement: pnad005_Supplementary_Data [file pnad005_supplementary_data.zip › pnad005_Supplementary_Data/UM SMART BACPAC Supplement 2 Deep Phenotyping_FINAL.pdf]

Supplement 2. Deep Phenotyping Visits (subset of 160 participants)

A subset of study participants (n = 160) be offered optional deep phenotyping. These participants will have a separate assessment appointment with study personnel at the CPFRC. There will be two visits that take place after T2-before Treatment 1 and after T3-before Treatment 2. At the deep phenotyping study visit, participants will undergo QST, structural and functional brain neuroimaging, inflammatory markers assessment, and the assessment autonomic functioning.

Quantitative Sensory Testing (QST)

Participants will complete a multimodal QST battery to characterize sensory function. Participant instructions are scripted, and all participants will undergo familiarization training for each procedure prior to data collection to reduce QST-related anxiety and measurement noise. All procedures can be stopped at any time by participant request if they become unbearable.

Pressure pain sensitivity will be assessed using an algometer with a 1-cm^2 rubber probe (FPX25, Wagner Instruments, Greenwich, CT, USA) to quantify pressure pain thresholds (PPT). PPT will be assessed at each participant's primary pain site, identified as the most painful area of the lumbar region in response to manual palpation. PPT will also be assessed at a remote control site located over the contralateral upper trapezius muscle (diagonal from lumbar site). Multisite pain hypersensitivity, particularly at asymptomatic remote sites, is a core feature of nociplastic pain.[1,2] Pressure will be manually increased at a rate of 0.5 kgf/cm^2/s (10 kgf max, metronome guided) until participants first indicate that the sensation pressure becomes one of faint pain. Pressure intensity (in kgf) at that time will be recorded as the PPT. Measurements will be conducted 3x/site (separated by 60-s rest intervals) with means used for analysis. Probe placement will be varied slightly trial to trial to prevent tissue sensitization from repeated testing of the same site. Additionally, we will use the Multimodal Automated Sensory Testing (MAST) System (Arbor Medical Innovations, LLC, Ann Arbor, MI, USA) [3-6] to apply computer-controlled pressure at the thumbnail bed to derive a suprathreshold measure of pressure intensity that evokes a moderate level of pain (i.e., Pain50) as well as pain tolerance. We have extensive experience using thumbnail pressure as an evoked pain stimulus and its validity in the measurement of nociplastic pain has been demonstrated extensively.[7-19] The system delivers an ascending series of discrete pressures (5-s duration; 4 kgf/cm^2/s ramp rate) at 20-s intervals, beginning at 0.50 kgf/cm^2 and increasing in 0.50 kgf/cm^2 steps. Pain intensity will be rated after each stimulus on a 0-100 numerical rating scale (NRS), with 0 being 'no pain' and 10 being 'worst pain imaginable.' The test will be terminated when participants reach their tolerance or 10 kgf/cm^2. Lastly, large volume, deep muscle pressure sensitivity[20,21] will be assessed using a MRI-compatible rapid cuff inflator (Hokanson, Bellevue, WA, USA).[22-24] This system includes an air compressor, computerized pressure controller, and a 13.5 cm X 82.5 cm velcro-adjusted pressure cuff. Participants will first receive an ascending series of cuff pressures, starting at 20 mmHG and increasing in 20 mmHG steps (10-s pressures, 20-s rest intervals) to personal tolerance or to a maximum of 400 mmHG. Each pressure will be rated after deflation on a 0-100 NRS. These pain ratings will be used to interpolate a series of 8 tolerable cuff pressures that will be delivered in pseudo-randomized order and rated individually on pain intensity and unpleasantness. Stimulus response curves will be constructed for each participant and used for analysis, along with several derived variables: *cuff-PPT, cuff-Pain50, and cuff-Tolerance*. In addition, tonic pain induced by continuous cuff pressure will be assessed (*tonic-Cuff)*. Each participant's individually calibrated Pain40 pressure (i.e., pressure that evokes a 40/100 pain

rating) will be applied for 6-min to one gastrocnemius muscle. Pain intensity and unpleasantness ratings will be obtained every 60-s.

Conditioned pain modulation (CPM) procedures require a conditioning-stimulus to induce endogenous analgesic systems and alter pain perception, and a test-stimulus to evaluate the endogenous analgesic response to the conditioning stimulus. CPM is attenuated in the majority of chronic pain participants and its magnitude is predictive of a variety of pain outcomes.[25-27] The CPM paradigm chosen for the UM-MRC was adopted from the method of Locke[31] and others,[32,33] and is consistent to that being used in the NIH Common Fund Acute to Chronic Pain Signatures network. In this procedure, immersion of one hand into a circulating 10°C cold water bath (NESLAB Digital One RTE 7, Thermo Scientific, Newington, NH, USA or similar) serves as the conditioning-stimulus and PPT at the trapezius serves as the test-stimulus. Baseline ratings of the test-stimulus will be acquired during the initial assessment of PPT (see above). Conditioning stimulation will begin by immersing the hand to a level 10 cm above the wrist into the water bath. The hand will be immersed for 60-s (or to each participant's individual tolerance); perceived pain of the water will be rated at 30-s and 60-s, or at the time of hand withdrawal, using a 0-100 NRS, to determine the adequacy of conditioning pain.[34] Immediately following hand withdrawal, PPT will be reassessed at the trapezius with 3 repetitions. CPM magnitude will be calculated as the difference in mean PPT measured prior to and after the conditioning stimulus, with increases in PPT following conditioning interpreted as evidence of endogenous pain inhibition.

Temporal Summation measures increases in excitatory pain pathways and is thought to reflect the progressive increase in dorsal horn neuronal firing in response to repetitive C-fiber stimulation.[35-38] Enhanced temporal summation is common in chronic pain and is predictive of pain outcomes.[39,40] We will evaluate temporal summation in triplicate (1-min rest intervals between trials) using a Neuropen fitted with a 40g Neurotip (Owen Mumford, Oxfordshire, United Kingdom). A train of 10 identical pinprick stimuli (applied at a rate 1 Hz, metronome guided) will be delivered separately to the primary pain site in the lumbar region and the volar forearm as a control site. After the 10th pinprick, the participant will be asked to rate the pain of the first and last pinprick using a 0-10 NRS. Participants will also rate any ongoing pain after-sensations at 15- and 30-s following the train of 10 stimuli. Temporal summation for each site will be calculated as the mean difference in pain ratings evoked by the first and last pinprick stimuli.

Michigan Visual Aversion Stress Test (M-VAST) will be used to probe mechanisms of sensory processing that bypass somatic peripheral receptors and the spinal cord, and are amplified in many chronic pain participants.[7,41-43] Participants will be presented with a flashing annular checkerboard pattern at varying illumination levels as performed previously.[44] Participants will be acclimated to a dark room and exposed to a high resolution, calibrated LED monitor displaying the visual stimulus. Each visual stimulus intensity level and the entire task will be rated on both sensory intensity and unpleasantness scales. These rating will be used to compute stimulus-response curves for analysis.

Two Point Discrimination Prior studies in participants with cLBP have documented subtle limitations in mechanical somatosensory sensitivity[45-47] and these are important to measure in a study of central nervous system processing of pain. Therefore, in addition to studying mechanical pain sensitivity, we will assess two-point discrimination thresholds, a measure of tactile acuity for non-painful mechanical sensation. As in previous studies, we will use a two-

point aesthesiometer (BASELINE Evaluation Instruments, White Plains, NY, USA), applied to the lumbar region and to a (non-painful) control site on the volar forearm. Participants will complete series of ascending (in which the 2 points of the aesthesiometer are initially adjacent) and descending (in which the 2 points of the aesthesiometer are initially far apart) trials in which they indicate whether they “feel one or two points” when the stimulus is applied. The distance between the points is then either increased or decreased until the experimenter locates the minimum distance at which the participant perceives 2 points instead of one. The results of ascending and descending trials are averaged to calculate the two-point discrimination threshold.

QST Feasibility and Anticipated Results These procedures engage different aspects of pain perception and potentially different peripheral and central mechanisms, thus permitting a comprehensive investigation into the psychophysical characteristics of cLBP. This testing strategy has been extensively employed and validated by our group and multisite networks, including the NIDDK MAPP[5,48] and LURN[48,49] Networks, and the German Neuropathic Pain Network.[50] We anticipate no significant issues with implementation. As stated above, we hypothesize that cLBP participants with lower pain sensitivity at the thumbnail (i.e., higher pain thresholds) will respond to acupressure,[51,52] whereas those with diminished CPM will be more likely to respond to duloxetine.[53] We further hypothesize that participants with multiple indices of nociplastic pain, including generalized pain hypersensitivity (i.e., increased pressure pain sensitivity at the lower back *and* remote body sites), facilitated temporal summation and visual hypersensitivity, will preferentially respond to centrally-acting treatments. Lastly, participants with localized pain hypersensitivity at the lower back, but not at other body areas, will show preferential response to interventional procedures.

Magnetic Resonance Imaging

All participants will also undergo spinal MRI to assess structural abnormalities of lower back (see Section 6.6 for imaging on all participants). Additionally, a subset of participants enrolled in the deep phenotyping study will undergo four different brain neuroimaging procedures (below): 1) functional connectivity MRI (fcMRI) at rest, 2) proton magnetic resonance spectroscopy (^{1}H-MRS) of the posterior insula assessing combined glutamate and glutamine (Glx), 3) evoked pressure pain at a neutral site (lower leg), and 4) high-resolution T1 structural imaging to assess gray matter volume in S1 and primary motor (M1) cortex. Following physical maneuvers to exacerbate clinical pain, resting state fcMRI and ^{1}H-MRS scans will be repeated to assess the neurobiological response to increases in pain, as we have previously demonstrated in FM using pressure.[54] The methods described below have been successfully employed previously by our group, and probe different aspects of the brain’s involvement in pain processing, thus complementing our QST methods by providing a comprehensive neurobiological signature of

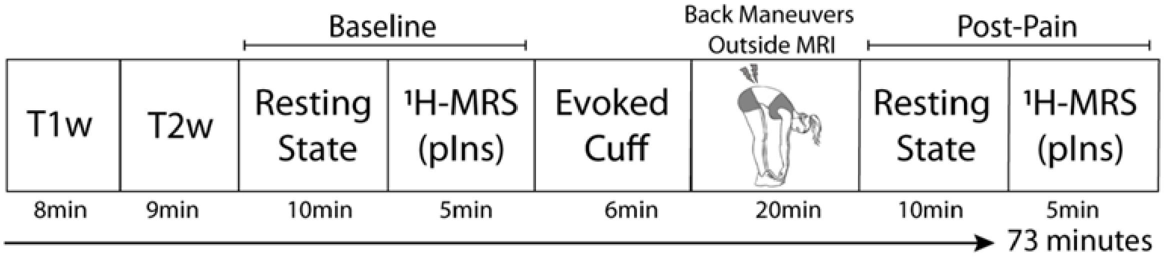

UM-MRC neuroimaging scanning session.

cLBP. Familiarization procedures will be completed prior to MRI to reduce anxiety. Neuroimaging will require approximately 90 minutes to complete. MRI will be done on a 3 Tesla GE scanner.

Resting State fcMRI. Brain imaging correlates of clinical pain have been notoriously difficult to measure;[55,56] however, our previous publications provide consistent evidence that our resting state fcMRI approach assays the neurocircuitry contributing to chronic pain as well as its modulation by pharmacological and non-pharmacologic interventions.[57-61] fcMRI is an adaptation of fMRI that examines intrinsic connectivity between brain regions - defined as ongoing synchronized neural activity occurring in the resting basal state. Intrinsic brain connectivity may be important for maintenance of synaptic connectivity and as such modulates the efficiency and extent of neuronal transmission between brain regions. Intrinsic connectivity, as measured by neuroimaging methods, follows known structural monosynaptic and polysynaptic pathways,[62,63] likely reflecting meaningful neurophysiological activity[64] within known primary sensory, executive, and associative networks.[65] fcMRI investigations are conducted with participants in an awake state, simply resting in the scanner. These data can then be analyzed with techniques such as independent component analysis (ICA), seed-voxel connectivity, and graph theoretical network techniques. While multiple resting state networks have been shown to be altered in chronic pain states, we will focus on three cardinal networks: the default mode network (DMN), the sensorimotor network (SMN), and the salience network (SLN). The DMN[65,66] is a constellation of brain regions engaged in self-referential cognition, which are 'deactivated' during various externally focused tasks. Our group's data in FM have found increased connectivity between the DMN and insula, a brain region thought to integrate the multiple dimensions of pain.[67] Greater clinical pain is correlated with greater DMN-insula connectivity[60], which is also diminished following treatment.[58,59] The SLN, containing the insula, is largely activated when one's attention is focused on specific external or internal tasks.[68] This network is altered in centralized pain participants, primarily showing increased connectivity between the SLN and S1/M1.[69]

Data Acquisition. Our group has published extensively using resting state connectivity outcomes in both cross-sectional participant-control[59,70,71] and longitudinal treatment trials.[57-59] In brief, participants will undergo two resting state fcMRI scans; one will be at the beginning of the scanning session and the second will immediately follow the evoked low back pain maneuvers. Ten minutes of resting state fMRI data will be acquired using a T2*-weighted multiband echo planar imaging (EPI) sequence (TR/TE:720/33 ms, flip angle (FA) = 52°, matrix size 90x90 with 60 slices encompassing whole brain, field of view (FOV) = 180 mm x 208 mm, 2.0x2.0x2.0 mm voxels and 833 volumes, MB factor 8). During the resting state fMRI participants will be instructed not to focus on any particular task and stay awake with their eyes open and focused on a fixation cross. Since cardiac and respiratory fluctuations are known to influence brain connectivity,[72] these data will be collected simultaneously using a chest plethysmograph and infrared pulse oximeter.

Preprocessing. fcMRI data are preprocessed using fMRIPrep (version 1.1.8)[73] running on the high-performance computing resources at our institution. Briefly, preprocessing steps include physiological noise removal (RETROICOR), motion correction, realignment, co-registration, normalization to standard MNI template, regression of nuisance variables (CompCor, motion parameters), and spatial smoothing (FWHM Gaussian kernel of 8mm).

Seed and Network (ICA) Connectivity Analysis. Seed to whole brain functional connectivity analysis is performed using the Conn (Cognitive and affective neuroscience laboratory, MIT, Cambridge, USA) functional connectivity toolbox. Seed regions (5 mm spheres) are chosen based on previously published fMRI studies of chronic pain, including: the insula,[61] and S1/M1.[69,74] Network seeds (DMN, SMN and SLN) will be derived from ICA, as previously published.[59,60,75] White matter, CSF, and motion parameters are used as covariates of no interest. Data is band pass filtered (0.01-0.1Hz) to remove linear drifts and high frequency noise from the data. First level analysis will correlate the time course from the seed/network to all brain voxels creating connectivity maps for each seed or network.

Group Level Analysis. Seed and network-to-whole-brain connectivity maps will be entered into a multiple regression model in SPM with change in pain interference (post minus pre-treatment) as a covariate of interest. Age and sex will be entered as covariates of no interest. Although not the primary objective of Aim 3, we will also conduct exploratory analyses to examine the mechanism of action of specific treatments. Changes in connectivity patterns following each of the phase 1 treatments will be performed using paired sample t-tests in SPM12. Resulting maps are thresholded at $p < .001$ uncorrected voxel threshold with $p \leq .05$ FDR significance corrected for multiple comparisons.

Graph Theory Analyses. Using Graph Theory Analyses the brain is defined as a network of 264 non-overlapping nodes (10 mm spheres) connected by edges or links.[76,77] These nodes and the preprocessed fcMRI data are entered into the Conn functional connectivity toolbox to create Fisher z-transformed bivariate correlation (Pearson's r) matrices (264 x 264) for each participant. To exclude weak or spurious connections, matrices are thresholded beginning with the strongest 5% connections and proceeding in steps of 5% up to 40% density, resulting in binary undirected graphs containing the most significant edges. Using the Brain Connectivity Toolbox,[78] we will calculate the following graph theoretical measures to assess global (efficiency, modularity, rich-club) and nodal (hub status) network properties, as previously published.[79] To assess hub status, we will calculate eigenvector centrality which takes into account the connectedness of a node, in addition to the connectedness of that node's neighbors. Hub status will be assigned to a node if the eigenvector centrality is greater than one standard deviation above the group mean.[80,81]

Group Level Analysis. We will determine if hub status and rich club organization predicts treatment response. Individual hub measures will be entered into regression analyses to predict ($p < .05$) association with change in pain interference pre- versus post-treatment.

Machine-learning based prediction analyses. SVM analyses will be performed for the previously mentioned modalities using the libsvm toolbox version 3.18[82] in Matlab 2017a and using group level images from the previously described modalities. Participant images will be labeled as a responder or non-responder based on change score of pain interference pre- versus post-treatment using a cutoff of a 30% reduction or a median split. SVM classification will be performed using a linear kernel with k-fold cross-validation to calculate classification accuracies. SVM model weights will be averaged across all cross-validation cases to investigate spatial distribution of the weights, with label permutation (n = X*1000) to establish significance levels.

Proton Magnetic Resonance Spectroscopy (1H-MRS*).* During ^{1}H-MRS, quantifiable measures of brain metabolites will be acquired from multiple brain regions non-invasively. The metabolite we will focus on is glutamate, the brain's major excitatory neurotransmitter. Previous

^{1}H-MRS studies, performed by our group, have examined changes in Glx (combined glutamate and glutamine) in cross sectional participant-control[18,51,83,84] and longitudinal treatment studies.[19,58] We find that FM participants display elevated Glx within the posterior insula and this concentration is reduced following active treatment, and this reduction was correlated with a reduction in clinical pain.[58] We have also shown that pelvic pain participants have elevated Glx within the anterior insula which is in turn associated with increased connectivity to the medial prefrontal cortex, a DMN structure.[85]

Data Acquisition. ^{1}H-MRS will be performed as in our previously published studies.[18,58,85] In brief, we will examine Glx within the posterior insula twice (before and after back pain maneuvers). Our primary ^{1}H-MRS outcome will be baseline Glx within the posterior insula. ^{1}H-MRS studies are performed on the same magnet as fcMRI. PRESS (TR/TE=2000/35 ms) single voxel ^{1}H-MRS is performed on the region of interest with voxel sizes of 2x2x3 cm. The water signal is recorded using 8 averages. Standardized voxel placements are guided by visual inspection of anatomical T1-weighted images as reported previously.[18]

Preprocessing. Raw data from each single-voxel MR spectroscopy sequence will undergo manual post-processing using ^{1}H-MRS software (LCModel; Stephen Provencher, Oakville, Ontario, Canada). LCModel uses a linear combination of individual spectra obtained from pure molecular species to fit the experimental spectra.[86] Glx values will be calculated both as an absolute concentration using the water signal for normalization and as a ratio to creatine. Glx absolute concentrations will be reported in arbitrary institutional units. Correction for cerebral spinal fluid (CSF) volume, which can dilute ^{1}H-MRS-derived Glx values, will be performed as reported previously.[18] Metabolite concentrations will be excluded if the Cramér-Rao bounds are greater than 20%.

Group Level Analysis. CSF-corrected Glx values will be entered into a multiple regression model in SPSS with change in pain (post minus pre-treatment) as the dependent variable and Glx values as a covariate of interest. Age and sex will be entered as nuisance regressors.

fMRI of Evoked Cuff Gastrocnemius Pain. Whole brain blood oxygen level-dependent (BOLD) functional images will be acquired using the same 3 Tesla scanner and T2*-weighted EPI multiband sequence as described for the resting state scan. Each participant will undergo a 11-minute block design scan, during which 6 cuff pressures (equal pressure = 15-30 and 100-115 mmHg; equal pain = 40-60/100 numerical rating scale (NRS) units will be applied to the left calf using the pressure cuff device (described above) in pseudo-random order. Pain levels for each participant will be determined during the QST assessment. Each pressure stimulus will be applied for 14 seconds. Prior to each pressure stimulus, a 4-second visual cue will be presented to signal upcoming cuff pressure to limit any stimulus-onset startle reflex. Participants' head motion will be minimized using foam pads around the head, and a strap secured across the forehead.

Pre-processing. Same as resting state fcMRI.

Group level analysis. The activation maps (pain minus rest) will be entered into a GLM to determine the neurobiological correlates of evoked pain that best predict treatment response (change in pain as the dependent variable). In addition, SVM will be implemented to create a machine learning model for prediction and discrimination between responder and non-responder. Resulting maps are thresholded at $p < .001$ uncorrected voxel threshold with $p \leq .05$ FDR significance corrected for multiple comparisons.

Physical Maneuvers to Evoke Back Pain. During their initial in-clinic visit, participants will perform simple exercise-like maneuvers (e.g., arching/bending the back to elicit lumbar flexion/extension) to evoke temporary increases in back pain. The experimenter will measure the degree to which a participant must flex or extend their back to evoke a clinical pain increase of at least 30% over their baseline. Detailed records will be taken of frequency and duration required to achieve the targeted clinical pain level to ensure the same maneuvers are performed at subsequent fMRI visits. For each participant, individualized physical maneuvers that are most reliable in exacerbating clinical pain will be picked for the fMRI sessions. During fMRI, participants will undergo baseline functional imaging (capturing brain activity at their baseline pain level), followed by a short (15-20 minute) break whereby participants will perform the physical maneuvers outside the scanner. Participants will re-enter the MRI environment and undergo post-exacerbation functional imaging (at their elevated pain level). Imaging analyses will focus on changes in brain activity within an individual (from baseline to exacerbated back pain). Participants will be cautioned to not increase their pain to a level that they are unable to tolerate. This procedure to exacerbate clinical back pain levels has been well-tolerated, feasible, and extensively used in past research,[87-89] however participants that are uncomfortable or concerned about performing this task can choose to skip it and complete the remainder of the fMRI session.

Autonomic Nervous System (ANS) acquisition during fMRI. During each fMRI scan, electrocardiogram (ECG), photoplethysmogram (PPG), respiration, skin temperature, and GSR data will be collected at 500 Hz using an MRI-compatible, noninvasive BIOPAC MP160 System (BIOPAC Systems, Goleta, CA, USA). For the ANS outcomes, ECG and respiratory volume signals will be acquired with 2 MR-compatible Ag/Ag-Cl transcutaneous sensors placed above and below the heart and a pneumatic belt placed just below the rib cage. GSR, PPG and temperature will be acquired using MR-compatible Ag/Ag-Cl transcutaneous sensors placed on the fingertips of the index and middle fingers.

Analysis. Inter-beat-intervals (segmented into 5-minute windows) and GSR signals (15-second epochs) will be imported into MATLAB for feature extraction. Windows with >15% missing data will be excluded. HRV features, including all time- and frequency-domain and Kubios features,[90] and tonic and phasic GSR features,[91] will be extracted using MATLAB. After feature extraction, we will utilize a semi-supervised machine learning paradigm[92] to combine the continuous HRV and GSR signals and characterize vagal tone.

Feasibility. We have previously collected ANS activity in participants undergoing QST and neuroimaging without difficulty. Risks associated with these measures are minimal since they are non-invasive. However, some participants may experience anxiety and/or a general unease associated with unfamiliar physiological testing and/or when applying or removing sensors. Participants will be instructed that they may stop participating at any time.

Brain structural Imaging to assess gray matter volume with Voxel Based Morphometry (VBM). We have published extensively using VBM methods in various chronic pain states.[93-96] In brief, our protocol includes T1-weighted structural images (TR 2400/TE 2.14ms; Flip angle 8 deg; FOV 224X224; Voxel size 0.8mm isotropic) segmented into white matter (WM), gray matter (GM), and cerebral spinal fluid using the segment function in SPM12, running under MATLAB 2017a. The resulting GM segments are then processed using the diffeomorphic anatomical registration through exponentiated lie algebra (DARTEL) toolbox.[97] We will normalize VBM data

are to standard space. We will smooth normalized, modulated images with a Gaussian kernel, and pass individual participant maps up to group level analysis.

Group Level Analyses: We will enter smoothed GM images into a multiple regression model in SPM12 with change in pain interference (post minus pre-treatment) as the covariate of interest. Age and total brain volume will be entered as nuisance regressors. We will exclude voxels with GM values < .1 from analysis.

Feasibility: We anticipate few problems in data analysis based on our past studies.[69,93-96]

Feasibility and Anticipated Neuroimaging Results. We have published extensively in the field of fcMRI, evoked pain fMRI, and ^{1}H-MRS, and expect no issues with data acquisition or analyses.[17-19,30,44,51,57-59,61,70,71,83,98-101]

Biospecimen Collection for Inflammation Methods

No more than 10mL of blood will be collected at each deep phenotyping visit. All samples will be stored initially at the University of Michigan but may be sent to other MRCs or central biorepositories for future storage or additional analyses.

Stimulated Assays. The TruCulture system (Myriad RBM) consists of small vacutainers preloaded with immune stimulants (e.g., LPS) or control media into which whole blood is drawn. Following incubation in a small table-top unit, the supernatant is isolated without the need for centrifugation using a small plunger included in the kit. Whole blood is drawn via venipuncture into two different 1 mL tubes containing lipopolysaccharide (LPS; 100 ng/mL), or media (NULL). LPS is potent agonist of TLR4,[102] Samples are immediately incubated at 37° C for 24 hours, after which the supernatant is isolated with a valve separator included in the kit. The supernatant is frozen at -80° C prior to shipment to a biorepository where it is thawed, aliquoted into 0.25 mL containers, and stored at -80° C for batch analysis. All batches include control standards (i.e., samples provided by lab personnel) to allow for estimation of batch effects.

Cytokine/Chemokines are analyzed from supernatant using multiplex Luminex xMap technology or equivalent. We have completed a series of dilution protocols to determine optimal concentrations to keep each analyte in the dynamic range of the assays. A minimum of seven cytokines/chemokines will be analyzed from each sample including those in the pro-inflammatory NF-κB- mediated suite (IL-1β, IL-6, TNF-α), chemotactic cytokines (IL-8, MCP-1, MIP1α), and regulatory cytokine IL-10.

Analyses. Cytokine/chemokine data often does not follow a normal distribution, which can create issues when using parametric statistical approaches. Therefore, we will evaluate normality with the Shapiro-Wilk test statistic and apply Box-Cox transformations where required. Any values below the lower limit of detection will be set to half that value. Multi-level models will be employed that can accommodate random effects for each batch analysis (in case significant variation is observed between-batches).

Feasibility and Anticipated Results. The protocol developed for MAPP serves as a template for this proposal because we have demonstrated that the procedures can be implemented with minimal training and without the need for dedicated wet lab space at the site of collection. In the second phase of MAPP, more than 95% of participant visits have resulted in analyzable samples across all three conditions. The stimulated assays are also clearly effective: in 198 samples from a preliminary MAPP II analysis, LPS-stimulated IL-6 showed a median increase of

21,000 pg/mL compared to media (unstimulated condition) while LPS-stimulated MIP1a showed a median increase of 48,000 pg/mL over media. We have also conducted a small analysis of freeze/thaw cycles as two such cycles are currently required for this protocol. After eight cycles, there was no evidence of change in analyte (e.g., IL-6, IL-1) levels.

1. Arendt-Nielsen L. Central Sensitization in Humans: Assessment and Pharmacology. In: Schaible H-G, ed. Berlin, Heidelberg: Springer Berlin Heidelberg; 2015:79-102.
2. Arendt-Nielsen L, Nie H, Laursen MB, et al. Sensitization in patients with painful knee osteoarthritis. *Pain.* 2010;149(3):573-581.
3. Harte SE, Mitra M, Ichesco EA, et al. Development and validation of a pressure-type automated quantitative sensory testing system for point-of-care pain assessment. *Med Biol Eng Comput.* 2013;51(6):633-644.
4. Wasserman RA, Hassett AL, Harte SE, et al. Pressure Pain Sensitivity in Patients With Suspected Opioid-Induced Hyperalgesia. *Reg Anesth Pain Med.* 2015;40(6):687-693.
5. Harte SE, Schrepf A, Gallop R, et al. Quantitative assessment of non-pelvic pain sensitivity in urological chronic pelvic pain syndrome: a MAPP Research Network study. *Pain.* In Press
6. Treister R, Lawal OD, Shecter JD, et al. Accurate pain reporting training diminishes the placebo response: Results from a randomised, double-blind, crossover trial. *PLoS One.* 2018;13(5):e0197844.
7. Geisser ME, Glass JM, Rajcevska LD, et al. A psychophysical study of auditory and pressure sensitivity in patients with fibromyalgia and healthy controls. *J Pain.* 2008;9(5):417-422.
8. Clauw DJ. Fibromyalgia and related conditions. *Mayo Clin Proc.* 2015;90(5):680-692.
9. Henry NL, Conlon A, Kidwell KM, et al. Effect of estrogen depletion on pain sensitivity in aromatase inhibitor-treated women with early-stage breast cancer. *J Pain.* 2014;15(5):468-475.
10. As-Sanie S, Harris RE, Harte SE, Tu FF, Neshewat G, Clauw DJ. Increased pressure pain sensitivity in women with chronic pelvic pain. *Obstet Gynecol.* 2013;122(5):1047-1055.
11. Giesecke J, Reed BD, Haefner HK, Giesecke T, Clauw DJ, Gracely RH. Quantitative sensory testing in vulvodynia patients and increased peripheral pressure pain sensitivity. *Obstet Gynecol.* 2004;104(1):126-133.
12. Gracely RH, Grant MA, Giesecke T. Evoked pain measures in fibromyalgia. *Best Pract Res Clin Rheumatol.* 2003;17(4):593-609.
13. Petzke F, Harris RE, Williams DA, Clauw DJ, Gracely RH. Differences in unpleasantness induced by experimental pressure pain between patients with fibromyalgia and healthy controls. *Eur J Pain.* 2005;9(3):325-335.
14. Petzke F, Clauw DJ, Ambrose K, Khine A, Gracely RH. Increased pain sensitivity in fibromyalgia: effects of stimulus type and mode of presentation. *Pain.* 2003;105(3):403-413.
15. Petzke F, Khine A, Williams D, Groner K, Clauw DJ, Gracely RH. Dolorimetry performed at 3 paired tender points highly predicts overall tenderness. *Journal of Rheumatology.* 2001;28(11):2568-2569.
16. Harris RE, Gracely RH, McLean SA, et al. Comparison of clinical and evoked pain measures in fibromyalgia. *J Pain.* 2006;7(7):521-527.
17. Gracely RH, Petzke F, Wolf JM, Clauw DJ. Functional magnetic resonance imaging evidence of augmented pain processing in fibromyalgia. *Arthritis Rheum.* 2002;46(5):1333-1343.
18. Harris RE, Sundgren PC, Craig AD, et al. Elevated insular glutamate in fibromyalgia is associated with experimental pain. *Arthritis Rheum.* 2009;60(10):3146-3152.

19. Harris RE, Sundgren PC, Pang Y, et al. Dynamic levels of glutamate within the insula are associated with improvements in multiple pain domains in fibromyalgia. *Arthritis Rheum.* 2008;58(3):903-907.
20. Izumi M, Petersen KK, Arendt-Nielsen L, Graven-Nielsen T. Pain referral and regional deep tissue hyperalgesia in experimental human hip pain models. *Pain.* 2014;155(4):792-800.
21. Graven-Nielsen T, Vaegter HB, Finocchietti S, Handberg G, Arendt-Nielsen L. Assessment of musculoskeletal pain sensitivity and temporal summation by cuff pressure algometry: a reliability study. *Pain.* 2015;156(11):2193-2202.
22. Loggia ML, Edwards RR, Kim J, et al. Disentangling linear and nonlinear brain responses to evoked deep tissue pain. *Pain.* 2012;153(10):2140-2151.
23. Polianskis R, Graven-Nielsen T, Arendt-Nielsen L. Modality-specific facilitation and adaptation to painful tonic stimulation in humans. *European Journal of Pain.* 2002;6(6):475-484.
24. Polianskis R, Graven-Nielsen T, Arendt-Nielsen L. Computer-controlled pneumatic pressure algometry--a new technique for quantitative sensory testing. *European Journal of Pain.* 2001;5(3):267-277.
25. Nir RR, Yarnitsky D. Conditioned pain modulation. *Curr Opin Support Palliat Care.* 2015;9(2):131-137.
26. Yarnitsky D. Role of endogenous pain modulation in chronic pain mechanisms and treatment. *Pain.* 2015;156 Suppl 1:S24-31.
27. O'Brien AT, Deitos A, Triñanes Pego Y, Fregni F, Carrillo-de-la-Peña MT. Defective Endogenous Pain Modulation in Fibromyalgia: A Meta-Analysis of Temporal Summation and Conditioned Pain Modulation Paradigms. *J Pain.* 2018;19(8):819-836.
28. Schoen CJ, Ablin JN, Ichesco E, et al. A novel paradigm to evaluate conditioned pain modulation in fibromyalgia. *J Pain Res.* 2016;9:711-719.
29. Neville SJ, Clauw AD, Moser SE, et al. Association Between the 2011 Fibromyalgia Survey Criteria and Multisite Pain Sensitivity in Knee Osteoarthritis. *Clin J Pain.* 2018;34(10):909-917.
30. Harper DE, Ichesco E, Schrepf A, et al. Resting Functional Connectivity of the Periaqueductal Gray Is Associated With Normal Inhibition and Pathological Facilitation in Conditioned Pain Modulation. *J Pain.* 2018;19(6):635 e631-635 e615.
31. Locke D, Gibson W, Moss P, Munyard K, Mamotte C, Wright A. Analysis of meaningful conditioned pain modulation effect in a pain-free adult population. *J Pain.* 2014;15(11):1190-1198.
32. Goodin BR, McGuire L, Allshouse M, et al. Associations between catastrophizing and endogenous pain-inhibitory processes: sex differences. *J Pain.* 2009;10(2):180-190.
33. Oono Y, Nie H, Matos RL, Wang K, Arendt-Nielsen L. The inter- and intra-individual variance in descending pain modulation evoked by different conditioning stimuli in healthy men. *Scandinavian Journal of Pain.* 2011;2(4):162-169.
34. Nir RR, Granovsky Y, Yarnitsky D, Sprecher E, Granot M. A psychophysical study of endogenous analgesia: the role of the conditioning pain in the induction and magnitude of conditioned pain modulation. *Eur J Pain.* 2011;15(5):491-497.
35. Graven-Nielsen T, Aspegren Kendall S, Henriksson KG, et al. Ketamine reduces muscle pain, temporal summation, and referred pain in fibromyalgia patients. *Pain.* 2000;85(3):483-491.
36. Price DD, Mao J, Frenk H, Mayer DJ. The N-methyl-D-aspartate receptor antagonist dextromethorphan selectively reduces temporal summation of second pain in man. *Pain.* 1994;59(2):165-174.
37. Price DD, Staud R, Robinson ME, Mauderli AP, Cannon R, Vierck CJ. Enhanced temporal summation of second pain and its central modulation in fibromyalgia patients. *Pain.* 2002;99(1-2):49-59.

38. Staud R, Vierck CJ, Cannon RL, Mauderli AP, Price DD. Abnormal sensitization and temporal summation of second pain (wind-up) in patients with fibromyalgia syndrome. *Pain.* 2001;91(1-2):165-175.
39. Petersen KK, Arendt-Nielsen L, Simonsen O, Wilder-Smith O, Laursen MB. Presurgical assessment of temporal summation of pain predicts the development of chronic postoperative pain 12 months after total knee replacement. *Pain.* 2015;156(1):55-61.
40. Weissman-Fogel I, Granovsky Y, Crispel Y, et al. Enhanced presurgical pain temporal summation response predicts post-thoracotomy pain intensity during the acute postoperative phase. *J Pain.* 2009;10(6):628-636.
41. Hollins M, Harper D, Gallagher S, et al. Perceived intensity and unpleasantness of cutaneous and auditory stimuli: an evaluation of the generalized hypervigilance hypothesis. *Pain.* 2009;141(3):215-221.
42. López-Solà M, Pujol J, Wager TD, et al. Altered functional magnetic resonance imaging responses to nonpainful sensory stimulation in fibromyalgia patients. *Arthritis Rheumatol.* 2014;66(11):3200-3209.
43. Martenson ME, Halawa OI, Tonsfeldt KJ, et al. A possible neural mechanism for photosensitivity in chronic pain. *Pain.* 2016;157(4):868-878.
44. Harte SE, Ichesco E, Hampson JP, et al. Pharmacologic attenuation of cross-modal sensory augmentation within the chronic pain insula. *Pain.* 2016;157(9):1933-1945.
45. Moseley GL. I can't find it! Distorted body image and tactile dysfunction in patients with chronic back pain. *Pain.* 2008;140(1):239-243.
46. Luomajoki H, Moseley GL. Tactile acuity and lumbopelvic motor control in patients with back pain and healthy controls. *Br J Sports Med.* 2011;45(5):437-440.
47. Wand BM, Di Pietro F, George P, O'Connell NE. Tactile thresholds are preserved yet complex sensory function is impaired over the lumbar spine of chronic non-specific low back pain patients: a preliminary investigation. *Physiotherapy.* 2010;96(4):317-323.
48. Clemens JQ, Mullins C, Ackerman AL, et al. Urologic chronic pelvic pain syndrome: insights from the MAPP Research Network. *Nat Rev Urol.* 2019;16(3):187-200.
49. Yang CC, Weinfurt KP, Merion RM, Kirkali Z. Symptoms of Lower Urinary Tract Dysfunction Research Network. *J Urol.* 2016;196(1):146-152.
50. Rolke R, Baron R, Maier C, et al. Quantitative sensory testing in the German Research Network on Neuropathic Pain (DFNS): standardized protocol and reference values. *Pain.* 2006;123(3):231-243.
51. Harte SE, Clauw DJ, Napadow V, Harris RE. Pressure Pain Sensitivity and Insular Combined Glutamate and Glutamine (Glx) Are Associated with Subsequent Clinical Response to Sham But Not Traditional Acupuncture in Patients Who Have Chronic Pain. *Med Acupunct.* 2013;25(2):154-160.
52. Zucker NA, Tsodikov A, Mist SD, Cina S, Napadow V, Harris RE. Evoked Pressure Pain Sensitivity Is Associated with Differential Analgesic Response to Verum and Sham Acupuncture in Fibromyalgia. *Pain Med.* 2017;18(8):1582-1592.
53. Yarnitsky D, Granot M, Nahman-Averbuch H, Khamaisi M, Granovsky Y. Conditioned pain modulation predicts duloxetine efficacy in painful diabetic neuropathy. *Pain.* 2012;153(6):1193-1198.
54. Ichesco E, Puiu T, Hampson JP, et al. Altered fMRI resting-state connectivity in individuals with fibromyalgia on acute pain stimulation. *Eur J Pain.* 2016;20(7):1079-1089.
55. Apkarian AV, Baliki MN, Geha PY. Towards a theory of chronic pain. *Prog Neurobiol.* 2009;87(2):81-97.
56. Tracey I. Imaging pain. *Br J Anaesth.* 2008;101(1):32-39.

57. Schmidt-Wilcke T, Ichesco E, Hampson JP, et al. Resting state connectivity correlates with drug and placebo response in fibromyalgia patients. *Neuroimage Clin.* 2014;6:252-261.
58. Harris RE, Napadow V, Huggins JP, et al. Pregabalin rectifies aberrant brain chemistry, connectivity, and functional response in chronic pain patients. *Anesthesiology.* 2013;119(6):1453-1464.
59. Napadow V, Kim J, Clauw DJ, Harris RE. Decreased intrinsic brain connectivity is associated with reduced clinical pain in fibromyalgia. *Arthritis Rheum.* 2012;64(7):2398-2403.
60. Napadow V, LaCount L, Park K, As-Sanie S, Clauw DJ, Harris RE. Intrinsic brain connectivity in fibromyalgia is associated with chronic pain intensity. *Arthritis Rheum.* 2010;62(8):2545-2555.
61. Ichesco E, Schmidt-Wilcke T, Bhavsar R, et al. Altered resting state connectivity of the insular cortex in individuals with fibromyalgia. *J Pain.* 2014;15(8):815-826 e811.
62. van den Heuvel MP, Mandl RC, Kahn RS, Hulshoff Pol HE. Functionally linked resting-state networks reflect the underlying structural connectivity architecture of the human brain. *Hum Brain Mapp.* 2009;30(10):3127-3141.
63. Krienen FM, Buckner RL. Segregated fronto-cerebellar circuits revealed by intrinsic functional connectivity. *Cereb Cortex.* 2009;19(10):2485-2497.
64. Raichle ME. A paradigm shift in functional brain imaging. *J Neurosci.* 2009;29(41):12729-12734.
65. Fox MD, Raichle ME. Spontaneous fluctuations in brain activity observed with functional magnetic resonance imaging. *Nat Rev Neurosci.* 2007;8(9):700-711.
66. Buckner RL, Vincent JL. Unrest at rest: default activity and spontaneous network correlations. *Neuroimage.* 2007;37(4):1091-1096; discussion 1097-1099.
67. Brooks JC, Tracey I. The insula: a multidimensional integration site for pain. *Pain.* 2007;128(1-2):1-2.
68. Menon V, Uddin LQ. Saliency, switching, attention and control: a network model of insula function. *Brain Struct Funct.* 2010;214(5-6):655-667.
69. Kutch JJ, Ichesco E, Hampson JP, et al. Brain signature and functional impact of centralized pain: a multidisciplinary approach to the study of chronic pelvic pain (MAPP) network study. *Pain.* 2017;158(10):1979-1991.
70. Hampson JP, Zick SM, Khabir T, Wright BD, Harris RE. Altered resting brain connectivity in persistent cancer related fatigue. *Neuroimage Clin.* 2015;8:305-313.
71. Darbari DS, Hampson JP, Ichesco E, et al. Frequency of Hospitalizations for Pain and Association With Altered Brain Network Connectivity in Sickle Cell Disease. *J Pain.* 2015;16(11):1077-1086.
72. Murphy K, Birn RM, Bandettini PA. Resting-state fMRI confounds and cleanup. *Neuroimage.* 2013;80:349-359.
73. Esteban O, Markiewicz CJ, Blair RW, et al. fMRIPrep: a robust preprocessing pipeline for functional MRI. *Nat Methods.* 2019;16(1):111-116.
74. Kong J, Spaeth RB, Wey HY, et al. S1 is associated with chronic low back pain: a functional and structural MRI study. *Mol Pain.* 2013;9:43.
75. Schrepf A, Kaplan CM, Ichesco E, et al. A multi-modal MRI study of the central response to inflammation in rheumatoid arthritis. *Nat Commun.* 2018;9(1):2243.
76. Cohen AL, Fair DA, Dosenbach NU, et al. Defining functional areas in individual human brains using resting functional connectivity MRI. *Neuroimage.* 2008;41(1):45-57.
77. Power JD, Cohen AL, Nelson SM, et al. Functional network organization of the human brain. *Neuron.* 2011;72(4):665-678.
78. Rubinov M, Sporns O. Complex network measures of brain connectivity: uses and interpretations. *Neuroimage.* 2010;52(3):1059-1069.
79. Kaplan CM, Schrepf A, Vatansever D, et al. Functional and neurochemical disruptions of brain hub topology in chronic pain. *Pain.* 2019.

80. Sporns O, Honey CJ, Kötter R. PLOS ONE: Identification and Classification of Hubs in Brain Networks. *PLoS ONE.* 2007.
81. van den Heuvel MP, Sporns O. Rich-club organization of the human connectome. *J Neurosci.* 2011;31(44):15775-15786.
82. Chang CC, Lin CJ. LIBSVM: A Library for Support Vector Machines. *Acm Transactions on Intelligent Systems and Technology.* 2011;2(3):1-27.
83. Foerster BR, Petrou M, Edden RA, et al. Reduced insular gamma-aminobutyric acid in fibromyalgia. *Arthritis Rheum.* 2012;64(2):579-583.
84. Petrou M, Pop-Busui R, Foerster BR, et al. Altered excitation-inhibition balance in the brain of patients with diabetic neuropathy. *Acad Radiol.* 2012;19(5):607-612.
85. As-Sanie S, Kim J, Schmidt-Wilcke T, et al. Functional Connectivity is Associated With Altered Brain Chemistry in Women With Endometriosis-Associated Chronic Pelvic Pain. *J Pain.* 2016;17(1):1-13.
86. Provencher SW. Automatic quantitation of localized in vivo 1H spectra with LCModel. *NMR Biomed.* 2001;14(4):260-264.
87. Lee J, Mawla I, Kim J, et al. Machine learning-based prediction of clinical pain using multimodal neuroimaging and autonomic metrics. *Pain.* 2019;160(3):550-560.
88. Wasan AD, Loggia ML, Chen LQ, Napadow V, Kong J, Gollub RL. Neural correlates of chronic low back pain measured by arterial spin labeling. *Anesthesiology.* 2011;115(2):364-374.
89. Loggia ML, Kim J, Gollub RL, et al. Default mode network connectivity encodes clinical pain: an arterial spin labeling study. *Pain.* 2013;154(1):24-33.
90. Tarvainen MP, Niskanen JP, Lipponen JA, Ranta-Aho PO, Karjalainen PA. Kubios HRV--heart rate variability analysis software. *Comput Methods Programs Biomed.* 2014;113(1):210-220.
91. Greco A, Valenca G, Scilingo E. *Advances in electrodermal activity processing with applications for mental health.* Cham, Switzerland: Springer International Publishing; 2016.
92. Lötsch J, Ultsch A. Machine learning in pain research. *Pain.* 2018;159(4):623-630.
93. Kairys AE, Schmidt-Wilcke T, Puiu T, et al. Increased brain gray matter in the primary somatosensory cortex is associated with increased pain and mood disturbance in patients with interstitial cystitis/painful bladder syndrome. *J Urol.* 2015;193(1):131-137.
94. Puiu T, Kairys AE, Pauer L, et al. Association of Alterations in Gray Matter Volume With Reduced Evoked-Pain Connectivity Following Short-Term Administration of Pregabalin in Patients With Fibromyalgia. *Arthritis Rheumatol.* 2016;68(6):1511-1521.
95. As-Sanie S, Harris RE, Napadow V, et al. Changes in regional gray matter volume in women with chronic pelvic pain: a voxel-based morphometry study. *Pain.* 2012;153(5):1006-1014.
96. Hsu MC, Harris RE, Sundgren PC, et al. No consistent difference in gray matter volume between individuals with fibromyalgia and age-matched healthy subjects when controlling for affective disorder. *Pain.* 2009;143(3):262-267.
97. Ashburner J. A fast diffeomorphic image registration algorithm. *Neuroimage.* 2007;38(1):95-113.
98. Hampson JP, Reed BD, Clauw DJ, et al. Augmented central pain processing in vulvodynia. *J Pain.* 2013;14(6):579-589.
99. Napadow V, Kettner N, Ryan A, Kwong KK, Audette J, Hui KK. Somatosensory cortical plasticity in carpal tunnel syndrome--a cross-sectional fMRI evaluation. *Neuroimage.* 2006;31(2):520-530.
100. Maeda Y, Kettner N, Holden J, et al. Functional deficits in carpal tunnel syndrome reflect reorganization of primary somatosensory cortex. *Brain.* 2014;137(Pt 6):1741-1752.
101. Harper DE, Ichesco E, Schrepf A, et al. Relationships between brain metabolite levels, functional connectivity, and negative mood in urologic chronic pelvic pain syndrome patients compared to controls: A MAPP research network study. *Neuroimage Clin.* 2018;17:570-578.
102. Lu YC, Yeh WC, Ohashi PS. LPS/TLR4 signal transduction pathway. *Cytokine.* 2008;42(2):145-151.
